# USP24-dependent stabilization of Runx2 recruits a p300/NCOA3 complex to transactivate *ADAMTS* genes and promote degeneration of intervertebral disc in chronic inflammation mice

**DOI:** 10.1186/s13062-023-00395-5

**Published:** 2023-07-06

**Authors:** Xingguo Li, Jun Zhang, Bing Wang, Chao Chen, Enyu Zhang, Zhengpin Lv, Qicong He, Yaoquan Hu, Xuenan Wang, Fan Zhang

**Affiliations:** Department of Orthopedics, The First Affiliated Hospital of Kunming Medical University, Kunming, Yunnan 650032 China

**Keywords:** USP24, ADAMTS, Runx2, p300, NCOA3, Chronic inflammation

## Abstract

**Background:**

Intervertebral disc degeneration (IDD) naturally occurs during the aging process. Its occurrence is closely related to chronic inflammation; however, the causal relationship between them is controversial. This study aimed to investigate if inflammation would promote IDD incidence and explore the underlying mechanism.

**Methods:**

A chronic inflammation mouse model was established by intraperitoneal injection of lipopolysaccharide (LPS). Enzyme-linked immunosorbent assay was performed to determine proinflammatory cytokines in serum. Histological staining was used to evaluate the degeneration of IVDs. Immunoblots and RT-qPCR analyses were performed to measure protein and mRNA expression levels. Immunoprecipitation, mass spectrometry, and co-immunoprecipitation assays were used to determine the assembly of protein complex.

**Results:**

We found that an inflammatory microenvironment activated p38 kinase, which phosphorylated the Runx2 transcription factor at the Ser28 site. The phosphorylated Runx2 (pRunx2) then recruited a deubiquitinase, ubiquitin-specific peptidase 24 (USP24), which stabilized pRunx2 and protected it from ubiquitin-dependent proteasomal degradation. The stabilized pRunx2 recruited histone acetyltransferase p300 and nuclear receptor coactivator 3 (NCOA3) to assemble a complex. This NCOA3-p300-pRunx2 complex then transactivated the expression of 13 *ADAMTS* (a disintegrin and metalloproteinase with thrombospondin motif) genes, thereby promoting the degradation of extracellular matrix (ECM) in intervertebral discs (IVDs) and causing IDD. Administration of either a p38 inhibitor (doramapimod), a NCOA3 inhibitor (bufalin), or a p300 inhibitor (EML425) significantly decreased the expression of the 13 *ADAMTS* genes and slowed the degeneration of IVDs.

**Conclusion:**

In summary, our results demonstrate that USP24 protects pRunx2 from proteasomal degradation under chronic inflammation conditions, enabling pRunx2 to transactivate *ADAMTS* genes and degrade ECM. Our findings provide direct evidence that chronic inflammation triggers IDD and offer a therapeutic strategy for retarding IDD in patients with chronic inflammation.

**Supplementary Information:**

The online version contains supplementary material available at 10.1186/s13062-023-00395-5.

## Introduction

Intervertebral disc degeneration (IDD) is a common musculoskeletal disorder in the elderly population [1, 2]. Approximately 85% of people will experience IDD in their lifetime, resulting in serious adverse effects on quality of life and even disabilities [3]. At present, the therapeutic strategies for patients with IDD are physical rehabilitation and surgical treatment/replacement of affected intervertebral discs (IVDs) [4]. Therefore, more in-depth studies of the pathogenic mechanisms of IDD will help us identify therapeutic strategies and develop effective medicines for IDD.

Each IVD consists of three consecutive parts: the inner nucleus pulposus (NP), the outer annulus (AF), and the cartilaginous endplates (EPs) that anchor the discs to adjacent vertebrae [5, 6]. With aging, IVDs undergo a series of morphological changes, including loss of height, annular tears, bulging, and osteophyte formation, which lead to IDD [7, 8]. Molecular investigations have revealed that IDD can be caused by multiple factors, such as DNA damage (caused by genotoxic stress, hyperosmolality, or nutritional stress), degradation of the extracellular matrix (ECM) in IVDs, chronic inflammation, cell death, cellular senescence, and oxidative stress [8–10].

ECM degradation in IVDs is mainly controlled by two classes of metalloproteinases: matrix metalloproteinases (MMPs) and a disintegrin and metalloprotease with thrombospondin motifs (ADAMTSs) [11, 12]. The human and mouse genomes encode 23 MMPs and 19 ADAMTSs [13]. These metalloproteinases show low expression in healthy IVDs but significantly elevated expression in degenerative IVDs [14, 15]. In humans and animals, degenerative IVDs show significantly elevated expression of 9 MMPs (MMP-1, -2, -3, -7, -8, -10, -12, -13, and − 14) and 7 ADAMTSs (ADAMTS-1, -3, -4, -5, -7, -12, and − 15); however, the regulatory mechanisms driving their upregulation are still largely unknown [14]. Recently, Tseng et al. revealed that runt-related transcription factor 2 (Runx2) recruited the CREB-binding protein (CBP) and PPARgamma coactivator 1alpha (PGC-1α) to transactivate *ADAMTS4/5* following stimulation of high doses of glucose [16]. The overexpressed ADAMTS4/5 promoted ECM degradation and caused IDD [16].

Emerging evidence now indicates that chronic inflammation is associated with the occurrence and development of IDD [1, 17]. Patients with IDD show significant increases in several proinflammatory cytokines, such as interleukin-1 beta (IL-1β), IL-6, IL-8, IL-17, and tumor necrosis factor-alpha (TNF-α) [1, 17]. Numerous studies have shown that IL-1β can induce *MMP* and *ADAMTS* genes at the transcriptional level [18, 19], but the specific mechanisms are still not clear. In the present study, we investigated whether chronic inflammation directly contributes to IDD through the transactivation of *ADAMTS* genes by establishing a chronic inflammation mouse model by intraperitoneal injection of a low dose (100 µg/kg) of lipopolysaccharide (LPS). We observed lumbar disc degeneration and upregulation of 13 *ADAMTS* genes (*ADAMTS-1*, -*4, -5, -6*, -*7, -8*, -*9*, -*14*, -*15*, -*16*, -*17*, -*18*, and − *20*) in LPS-treated mice. We also found that ubiquitin-specific peptidase 24 (USP24) protected p38-phosphorylated Runx2 (pRunx2) from ubiquitin-dependent proteasomal degradation, thereby enabling pRunx2 to recruit two transcriptional regulators, namely p300 (a histone acetyltransferase) and the nuclear receptor coactivator 3 (NCOA3), to assemble a NCOA3-p300-pRunx2 complex. This transcriptional complex specifically bound to the promoters of *ADAMTS-1*, -*4, -5, -6*, -*7, -8*, -*9*, -*14*, -*15*, -*16*, -*17*, -*18*, and − *20* and transactivated their expression. Our results provide direct evidence confirming chronic inflammation as an important trigger of IDD. Blockage of the formation of the pRunx2-p300-NCOA3 complex may therefore represent a new therapeutic strategy for preventing IDD.

## Materials and methods

### Animal experiments

All animal experiments were performed in accordance with a protocol (2019IDD-043) that was reviewed and approved by the Ethics Committee of Kunming Medical University. Briefly, eight-week old C57BL/6 mice (male, 23–25 g; Charles River; Shanghai, China) housed in a specific pathogen-free (SPF) room at the animal facility of the First Affiliated Hospital of Kunming Medical University were randomly assigned to a sham or an LPS group (n = 8 for each group). The sham group mice were intraperitoneally injected with 100 µL phosphate-buffered saline (PBS) (pH7.4) (Sigma-Aldrich; Shanghai, China; #806,552). The LPS group mice were intraperitoneally injected with 100 µg/kg LPS (Sigma-Aldrich; #L5293). Injections in both groups were performed at 7 day intervals for 4 weeks. The LPS-challenged mice were then randomly divided into four subgroups and injected with either 100 µL PBS (LPS-sham group), the p300 inhibitor EML425 (15 mg/kg; MedChemExpress; Monmouth Junction, NJ, USA; # HY-110,263) (EML425 group), the p38 inhibitor doramapimod (4 mg/kg; Selleck Chemicals; Houston, TX, USA; #S1574) (doramipimod group), or the NCOA3 inhibitor bufalin (1 mg/kg; Selleck Chemicals; #S7821) (bufalin group). The injections were performed at 7-day intervals for a further 6 weeks.

At the end of the 10 week treatment period, all mice were anesthetized using 3% isoflurane via inhalation and maintained at 0.5% isoflurane. Lumbar discs were recorded using magnetic resonance imaging (MRI) following a previous protocol [16]. After MRI imaging, the mice were sacrificed using an overdose of inhaled CO_2_ and blood samples were immediately collected and stored in 1.5 mL Eppendorf tubes. Serum was obtained by centrifuging blood samples at 2000 *g* for 10 min. Whole IVDs and NP/AF tissues were separated as described previously [20].

### Determination of proinflammatory cytokines by enzyme-linked immunosorbent assay (ELISA)

Mouse serum samples were used to measure the proinflammatory cytokines IL-1β, IL-6, IL-15, IL-17, and TNF-α. The following ELISA kits were used: IL-1β (Thermo Fisher Scientific; Shanghai, China; #BMS6002), IL-6 (Thermo Fisher Scientific; #KMC0061), IL-15 (Thermo Fisher Scientific; #900-M188), IL-17 (Thermo Fisher Scientific; #BMS6001), and TNF-α (Thermo Fisher Scientific; #BMS607HS). The measurements were performed following the manufacturer’s guidelines.

### Histological staining

Histological staining was performed following a previous method [16]. Briefly, the L1/L2 lumbar IVDs were isolated from the different mouse groups (LPS-Sham, LPS, EML425, doramapimod, and bufalin) of mice. The isolated IVDs were fixed with 10% neutral buffered formalin (Sigma-Aldrich; # HT501128) for 24 h and decalcified with a mild decalcifier solution (Osteosoft; Sigma-Aldrich; #1,017,289,010) for 72 h. The samples were then dehydrated in ethanol, embedded in paraffin, and cut into Sect. 10 μm thick. After deparaffinizing and rehydrating, the sections were stained with 1% Alcian blue solution (Sigma-Aldrich; #B8438), 3 mL glacial acetic acid (Sigma-Aldrich; #1,371,301,000), and 97 mL distilled water (ddH_2_O) for 30 min. The sections were rinsed in ddH_2_O for 2 min, stained with 0.02% fast green (Sigma-Aldrich; #F7252) for 1 min, and incubated in 100 mL 1.3% picric acid solution (Sigma-Aldrich; #P6744) for 1.5 h. The sections were then stained with 1.0% safranin O (Sigma-Aldrich; #S2255) for 30 min, rinsed with 0.01 N HCl for 4 min, dehydrated, cleared, mounted, and coverslipped. The sections were photographed using an inverted TE 2000 wide-field microscope system (Nikon).

### Isolation, culture, and transfection of primary mouse NP and AF cells

Primary NP and AF cells were isolated from the lumbar discs of three C57BL/6 mice following previously described protocols [20]. The obtained cells, which were designated as NP-1, NP-2, NP-3, AF-1, AF-2, and AF-3 cells, were cultured in Dulbecco’s Modified Eagle Medium (DMEM)/Nutrient Mixture F-12 Ham (Sigma-Aldrich; #D8437) supplemented with 10% (v/v) fetal bovine serum (FBS) (Sigma-Aldrich; #F2442) and 1 × penicillin-streptomycin (Sigma-Aldrich; #P4458).

Cells at 80% confluence were transfected with 1 µg shRNA plasmid DNA targeting specific genes (Table S1) or 1 µg overexpression plasmid DNA (Table S2), following a previous protocol [16]. The transfected cells were cultured in an antibiotic-free medium for 24 h, then the medium was aspirated and replaced with fresh medium containing 2 µg/mL of puromycin for selection. Individual puromycin-resistant cells were harvested and subjected to RNA and protein isolation to verify the mRNA and protein expression levels of the targeted genes.

### In vitro cell treatments

For LPS-only treatment, cells at 80% confluence were treated with 20 ng/mL LPS (Sigma-Aldrich; #L4516) for 6 h, washed twice with ice-cold PBS, and then subjected to RNA and protein isolation. For the co-treatments with LPS and p38, NCOA3, or p300 inhibitors, the cells were simultaneously treated with 20 ng/mL LPS and either 1 µM EML425 (p300 inhibitor), 40 nM doramapimod (p38 inhibitor), or 10 nM bufalin (NCAO3 inhibitor) for 6 h, followed by two washings with ice-cold PBS and then RNA and protein isolation.

### RNA isolation and quantitative reverse transcription PCR (RT-qPCR)

RNA was isolated from cells (5 × 10^6^) and tissues (0.1 g) using TRIzol (Thermo Fisher Scientific; #15,596,026) and following the manufacturer’s guidelines. A 1 µg sample of total RNA was reverse transcribed into first-strand cDNA using LunaScript RT SuperMix (New England Biolabs; Shanghai, China; #E3010L). The obtained cDNAs were diluted 100-fold and then used for RT-qPCR analyses to detect gene expression levels with the PowerTrack SYBR Green Master Mix (Thermo Fisher Scientific; #A46109). Relative gene expression levels were quantified using the 2^−ΔΔCt^ method in which ΔΔCt = Ct_gene_-Ct_β−Actin_. The primers used for RT-qPCR analyses are listed in Table S3.

### Protein extraction and western blotting

Proteins from cells (5 × 10^6^) and tissues (0.1 g) were extracted in radioimmunoprecipitation assay (RIPA) buffer (Thermo Fisher Scientific; #89,900), and 50 µg samples of total protein from each sample were separated on 12% SDS-PAGE gels, followed by transfer to polyvinylidene difluoride (PVDF) membranes (Thermo Fisher Scientific; #88,518). After incubation with 5% non-fat milk (Santa Cruz Biotechnology; Shanghai, China; #sc2324), the PVDF membranes were probed with primary antibodies (Table S4) and secondary antibodies (Table S4). Protein signals were visualized using an enhanced chemiluminescence (ECL) reagent (Thermo Fisher Scientific; #32,106).

### Immunoprecipitation (IP), mass spectrometry (MS), and co-immunoprecipitation (Co-IP) assays

Equal weights (0.1 g) of three individual IVDs from the LPS-treated mice were combined and homogenized in 1 mL RIPA buffer containing 1 × Protease and Phosphatase Inhibitor Cocktail (Thermo Fisher Scientific; #78,440). The cell lysates were centrifuged at 15,000 *g* for 15 min to remove cell debris. The supernatants were divided into two equal parts and immunoprecipitated with either anti-Runx2-coated or IgG-coated protein A agarose (Thermo Fisher Scientific; #20,333). The Runx2- and IgG-associated proteins were separated on 12% SDS-PAGE gels and the protein bands were visualized using the Pierce Silver Stain Kit (Thermo Fisher Scientific; #24,612). The protein bands were then destained and in-gel digested with trypsin (Sigma-Aldrich; #11,418,475,001) overnight at 37 °C. The resulting peptides were sequentially purified using 5% formic acid (Sigma-Aldrich; #1,002,631,000)/50% acetonitrile (Sigma-Aldrich; #34,851) and 0.1% formic acid/75% acetonitrile. The eluted peptides were concentrated to 20 µL, followed by analysis with an LTQ ORBITRAP Velos mass spectrometer (Thermo Fisher Scientific). The results were analyzed by performing data searches on an in-house Mascot server (Matrix Science, London, UK) against the International Protein Index in the mouse protein database.

Direct interaction between two proteins was determined by lysing cells expressing different combinations of Flag-tagged + MYC-tagged vectors in RIPA buffer. The cell lysates were immunoprecipitated by exposure to anti-Flag agarose (Sigma-Aldrich; #A4596) and anti-MYC-agarose (Thermo Fisher Scientific; #20,169) at 4 °C for 2 h. The agarose beads and their bound proteins were washed 5 times with RIPA buffer supplemented with 1 × Protease and Phosphatase Inhibitor Cocktail. The input and output proteins were separated on 12% SDS-PAGE gels, and anti-Flag and anti-MYC were then used to determine the protein levels (Table S4).

### Chromatin immunoprecipitation (ChIP) assays

Cells (5 × 10^7^) were crosslinked with 1% (w/v) formaldehyde (Sigma-Aldrich; #252,549) for 10 min. The crosslinking reaction was terminated by adding glycine to a final concentration of 125 mM and incubating for 5 min at room temperature. The crosslinked cells were lysed in ChIP lysis buffer (Santa Cruz Biotechnology; #sc45000) and chromatin was fragmented by sonication to an average length of 300–500 bp. Cell debris was removed by centrifuging at 15,000 *g* for 15 min, and the supernatants were used for immunoprecipitation by treating with anti-Runx2-, anti-pRunx2^S28^, anti-p300-, anti-NCOA3-, and IgG-coated protein A agarose at 4 °C for 3 h. The input and output DNAs were isolated and used as templates for ChIP-RT-qPCR assays with the primers listed in Table S5.

### Deubiquitination assay

For the in vivo deubiquitination assay, NP-1 cells were infected with HA-Ubiquitin, shUSP24 (Sigma-Aldrich; #TRCN0000040628) + HA-Ubiquitin, or shUSP24 + MYC-USP24 + HA-Ubiquitin. After 48 h, cells were treated with 20 ng/mL LPS for 6 h. Subsequently, the cells were collected for IP assay using anti-Runx2^Ser28^ (Thermo Fisher Scientific; #PA5-105643), followed by a western blotting assay to detect ubiquitination. For the in vitro deubiquitination assay, the immunoprecipitated Runx2^Ser28^ from LPS-treated NP-1 cells expressing shUSP24 + HA-Ubiquitin were incubated with various amounts (0, 0.01, 0.02, 0.04, and 0.08 nM) of recombinant USP24 (OriGene; Wxi, Jiangsu, China; #TP526420) in a deubiquitination buffer composed of 50 mM Tris-HCl (pH 7.5) and 10 mM DTT at 37 °C for 1 h. The reaction was halted and resolved using SDS-PAGE loading buffer, followed by immunoblotting with the anti-HA antibody (Sigma-Aldrich; #11,583,816,001).

### Statistical analysis

All experiments were independently repeated in triplicate. All data were presented as means ± standard deviation (SD). Statistical analyses comparisons between groups were performed using one-way ANOVA coupled with Tukey’s post-hoc test and Statistical Package for the Social Sciences (SPSS) software (IBM, NY, USA; version 18). *P* < 0.05 was considered statistically significant. One asterisk (*) represents *P* < 0.05, two asterisks (**) represent *P* < 0.01, and three asterisks (***) represent *P* < 0.001.

## Results

### Thirteen ***ADAMTS*** genes were upregulated in the IVDs of chronic inflammation mice and in low-dose LPS-treated NP and AF cells

**Fig. 1:**
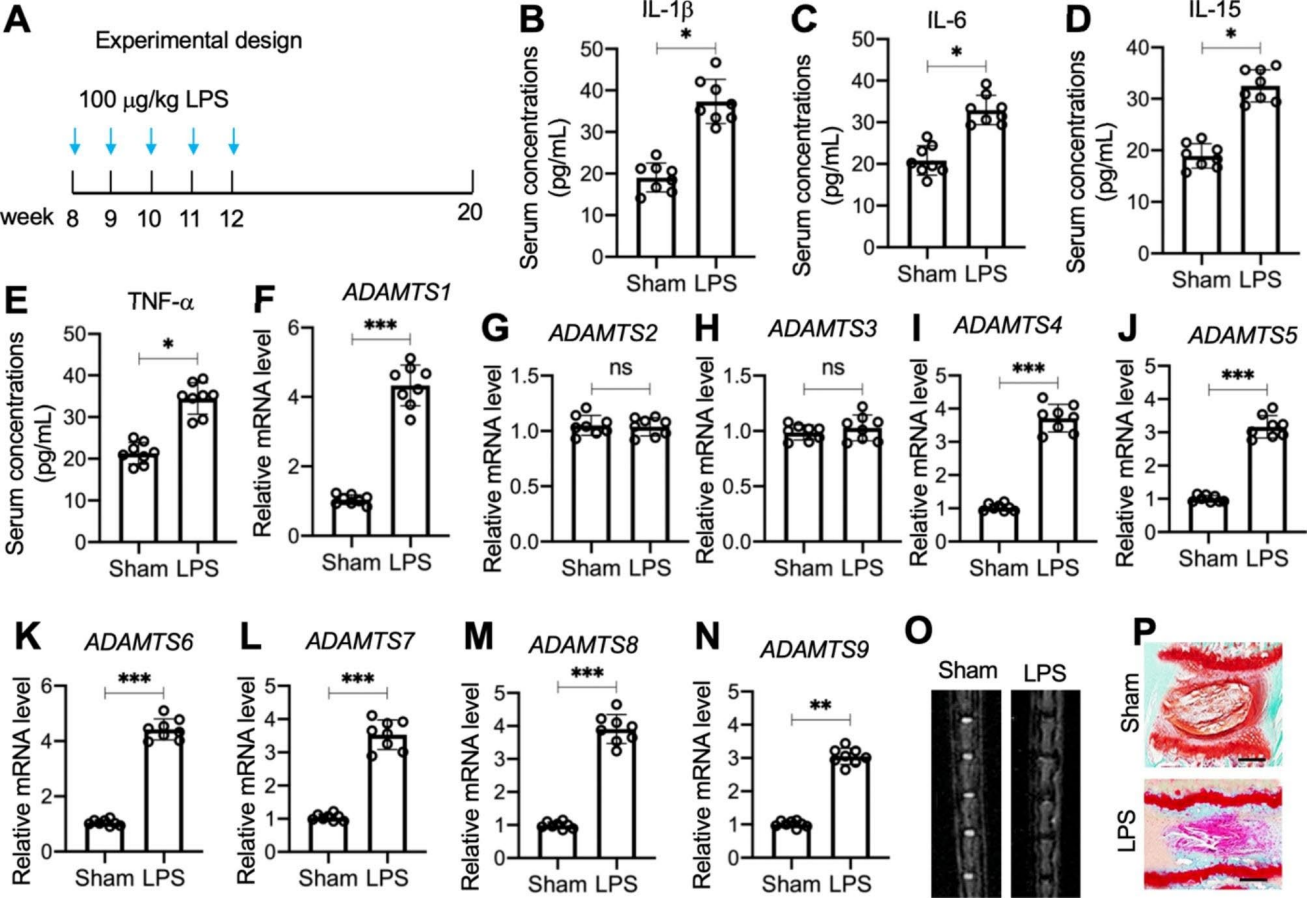
Chronic inflammation promoted the degeneration of IVDs and induced *ADAMTS* levels in LPS-challenged mice **(A)** Time points of LPS injection. **(B-E)** Serum concentrations of proinflammatory cytokines: IL-1β **(B)**, IL-6 **(C)**, IL-15 **(D)**, and TNFα **(E). (F-N)** The mRNA expression levels of *ADAMTS* genes in sham- and LPS-treated mice (n = 8). **(F)***ADAMTS1*; **(G)***ADAMTS2*; **(H)***ADAMTS3*; **(I)***ADAMTS4*; **(J)***ADAMTS5*; **(K)***ADAMTS6*; **(L)***ADAMTS7*; **(M)***ADAMTS8*; **(N)***ADAMTS9*. **(O)** Representative MRI images of lumbar IVDs from sham- and LPS-treated mice. **(P)** Representative picrosirius red staining images of IVDs from sham- and LPS-treated mice. Bars = 100 μm. *P < 0.05; **P < 0.01; ***P < 0.001; ns: no significant difference

We investigated whether chronic inflammation directly caused IDD through an upregulation of *ADAMTS* genes by intraperitoneally injecting 8-week-old C57BL/6 mice (n = 8) with 100 µg/kg LPS at a one-week interval for four weeks (Fig. 1A). Eight weeks later, we observed mildly elevated levels of proinflammatory cytokines (IL-1β, IL-6, IL-15, and TNF-α) in mouse serum (Fig. 1B and E). We also detected all 19 *ADAMTS* genes in sham-IVDs and LPS-IVDs; 13 (*ADAMTS-1*, -*4, -5, -6*, -*7, -8*, -*9*, -*14*, -*15*, -*16*, -*17*, -*18*, and − *20*) were upregulated in the degenerative IVDs (Fig. 1F N and S1). MRI images and histological results (safranin O and fast green staining) confirmed significant degeneration in the lumbar IVDs of LPS-treated mice but not in the sham mice (Fig. 1O and P).

We sought to clarify whether the upregulation of *ADAMTS* genes was caused by LPS-induced chronic inflammation by treating primary NP (#1 and #2) and AF (#1 and #2) cells with a low dose of LPS (20 ng/mL) for 6 h. As in the LPS-treated mice, 13 *ADAMTS* genes (*ADAMTS-1*, -*4, -5, -6*, -*7, -8*, -*9*, -*14*, -*15*, -*16*, -*17*, -*18*, and − *20*) were induced in the LPS-treated NP and AF cells (Figures S2 and S3). The other 6 *ADAMTS* genes (*ADAMTS*-*2, -3*, -*10*, -*12*, -*13*, and − *19*) were not induced by LPS (Figures S2 and S3).

### Runx2 mediated the expression of ***ADAMTS*** genes in NP/AF cells

We explored possible mechanisms for the differential expression of *ADAMTS* genes in degenerative IVDs by first analyzing the promoters of *ADAMTS* genes, with the aim of identifying transcription factors controlling *ADAMTS* expression levels. We found that multiple transcription factors, including nuclear factor kappa B (NF-κB), transcription factor 4 (TCF4), and Runx2, could bind to the promoters of different *ADAMTS* genes (Figure S4). Interestingly, Runx2, but not the other transcription factors, was predicted to bind to the promoters of the 13 *ADAMTS* genes we found overexpressed in degenerative IVDs (Figure S4). Thus, we speculated that Runx2 might be the key transcription factor that controls *ADAMTS* expression in IVD caused by chronic inflammation.

**Fig. 2:**
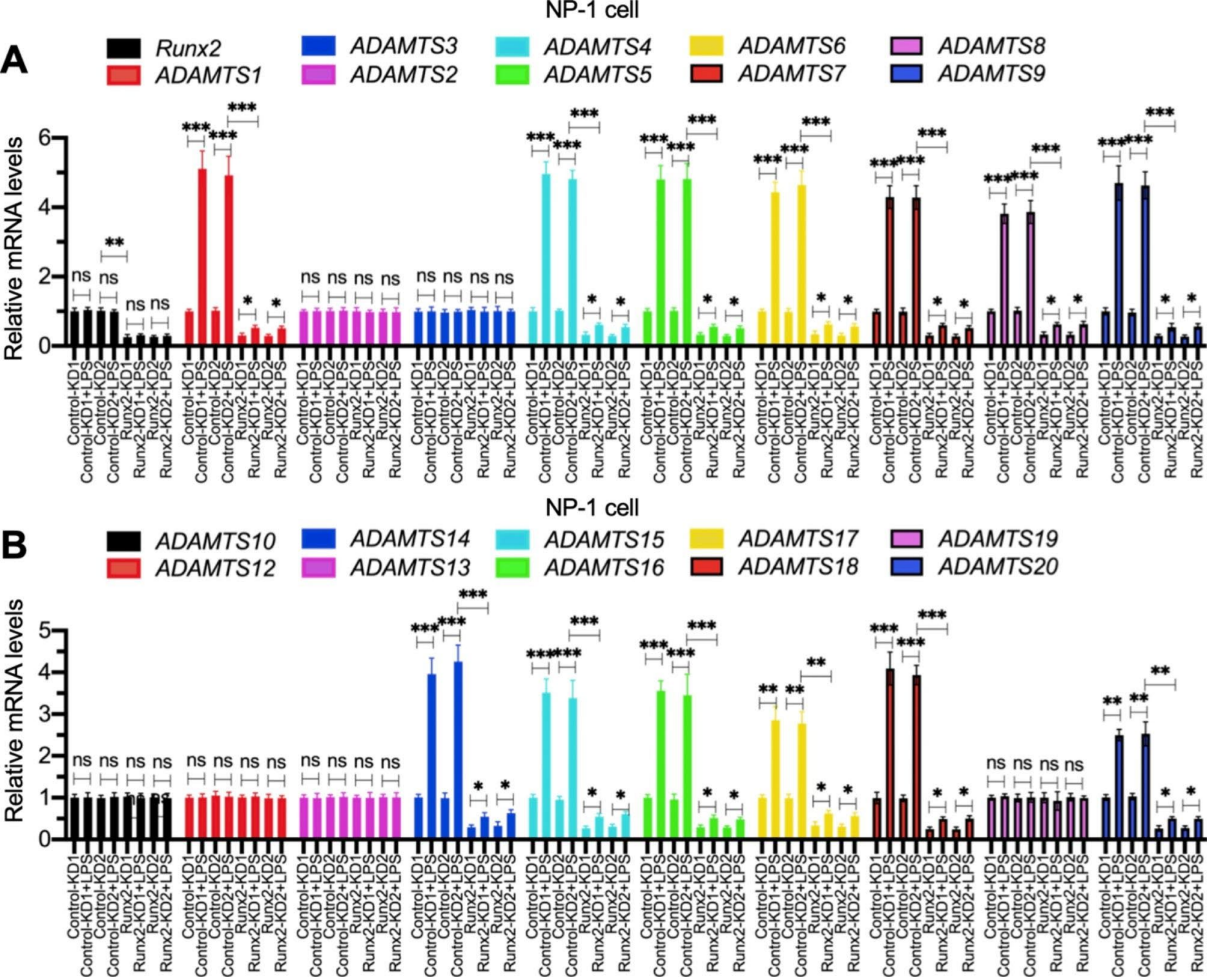
The effects of Runx2 depletion on the expression of *ADAMTS* genes in NP-1 cells treated with or without LPS The Control-KD1/2 and Runx2-KD1/2 cells in the NP-1 background were treated with or without 20 ng/mL LPS for 6 h, followed by RNA isolation and RT-qPCR analyses to examine the mRNA levels of *Runx2* and *ADAMTS* genes. **(A)***Runx2*, *ADAMTS1*, *-2*, *-3*, *-4*, *-5*, *-6*, *-7*, *-8*, and *− 9*. **(B)***ADAMTS10*, -*12*, *-13*, *-14*, *-15*, *-16*, *-17*, *-18*, *-19*, and *− 20*. *P < 0.05; **P < 0.01; ***P < 0.001; ns: no significant difference

RT-qPCR results for two independent Runx2-KD cell lines, constructed from NP-1 and AF-1 cells, showed that the depletion of Runx2 decreased the expression of *ADAMTS-1*, -*4, -5, -6*, -*7, -8*, -*9*, -*14*, -*15*, -*16*, -*17*, -*18*, and − *20* but did not affect the expression of *ADAMTS*-*2, -3*, -*10*, -*12*, -*13*, and − *19* in both cell backgrounds (Fig. 2 and S5). By contrast, the induction of the 13 *ADAMTS* genes in LPS-treated NP-1/AF-1 cells was significantly suppressed by Runx2-depletion (Fig. 2 and S5), suggesting that Runx2 mediated the expression of *ADAMTS* genes in NP/AF cells.

### Runx2 recruited p300 and NCOA3 to assemble a transcriptional complex

**Fig. 3:**
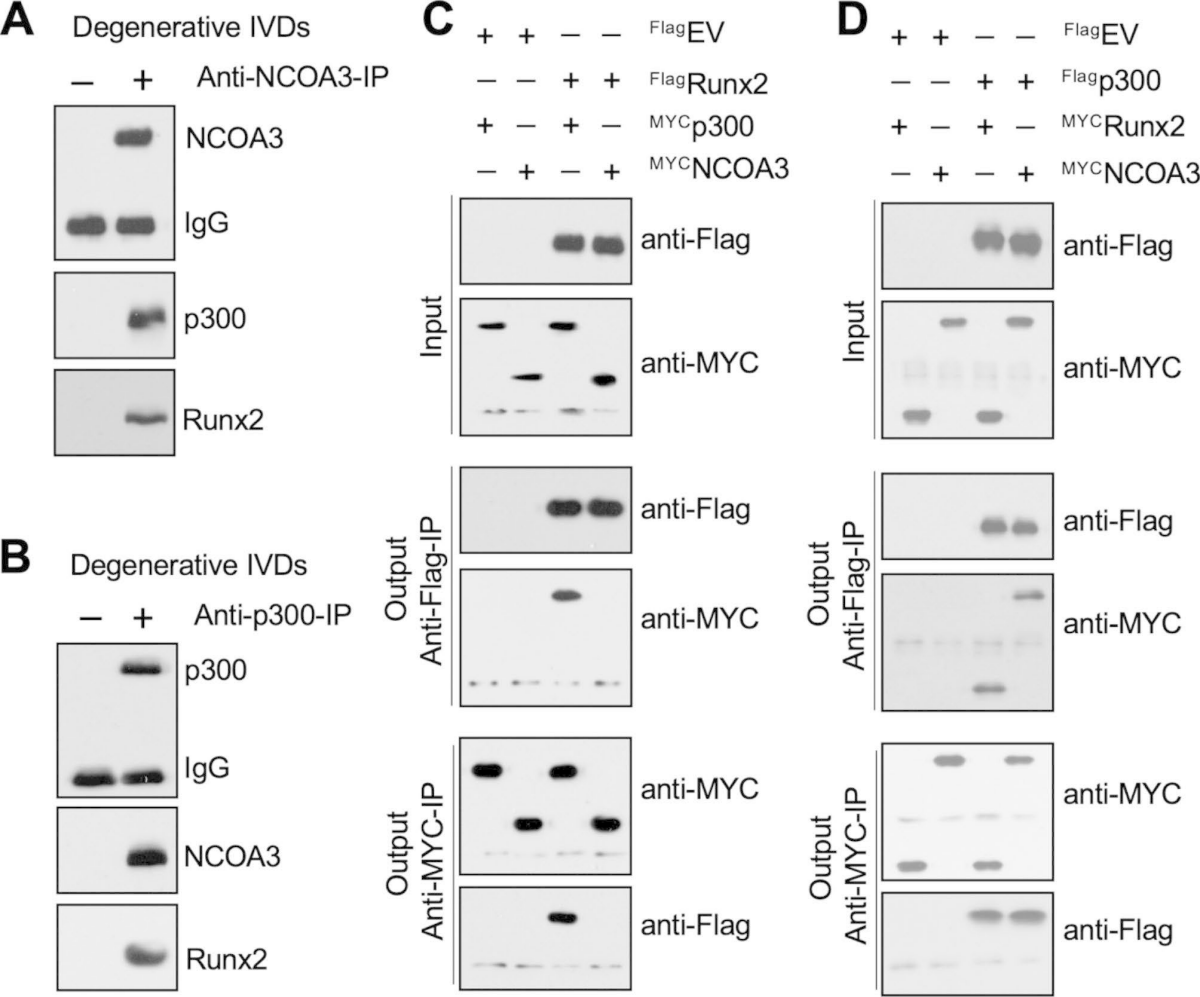
Runx2 recruited p300 and NCOA3 to assemble a complex in vivo and in vitro **(A)** NCOA3 pulled down both Runx2 and p300 in vivo. Equal weights (0.05 g) of three independent IVDs from LPS-challenged mice were mixed to make a homogenate, followed by immunoprecipitation with anti-NCOA3- and IgG-coated protein A agarose. The purified complexes were used for western blotting assays with anti-NCOA3, anti-Runx2, and anti-p300 antibodies. **(B)** p300 pulled down both Runx2 and NCOA3 in vivo. The same homogenate used in (A) was immunoprecipitated with anti-p300- and IgG-coated protein A agarose. The purified complexes were used for western blotting assays with anti-P300, anti-Runx2, and anti-NCOA3 antibodies. **(C** and **D)** In vitro Co-IP results. Different combinations of Myc-tagged and Flag-tagged plasmids, as shown in the figure, were co-transfected into NP-1 cells. After incubation at 37 °C for 48 h, cells were used for immunoprecipitation using anti-Flag-agarose. The input and output proteins were detected with anti-Flag and anti-Myc antibodies. **(C)** Determination of the direct interaction of ^Flag^Runx2-^Myc^p300 and ^Flag^Runx2-^Myc^NCOA3. **(D)** Determination of the direct interaction of ^Flag^p300-^Myc^Runx2 and ^Flag^p300-^Myc^NCOA3.

We next used degenerative IVDs from LPS-treated mice (n = 3) to perform IP assays using anti-Runx2- and IgG-coated agarose beads. The bands showing positive silver staining were then used for mass spectrometry analyses to identify Runx2-interacting proteins, and we obtained 42 candidate proteins (Table S6. A search for transcriptional regulators among these candidates revealed p300 and NCOA3 (Table S6). We then verified that Runx2 interacted with NCOA3 and p300 by conducting two IP experiments using anti-NCOA3-coated and anti-p300-coated agarose in degenerative IVDs from LPS-treated mice (n = 3). Both NCOA3 and p300 pulled down Runx2 (Fig. 3A and B), suggesting that Runx2, p300, and NCOA3 formed a complex in vivo.

We determined how Runx2, p300, and NCOA3 assembled into a complex by cotransfecting NP-1 cells with ^Flag^Runx2+^MYC^P300, ^Flag^Runx2+^MYC^NCOA3, ^Flag^P300+^MYC^Runx2, and ^Flag^P300+^MYC^NCOA3. Co-IP assays with anti-Flag-agarose and anti-MYC-agarose revealed a direct interaction between Runx2 and p300, but not between Runx2 and NCOA3 (Fig. 3C). By contrast, p300 could directly interact with both Runx2 and p300 (Fig. 3D). These results suggested that Runx2 might first recruit p300 to form a dimer that can then interact with NCOA3 to form a functional transcriptional complex.

### Runx2 was phosphorylated by p38 kinase in LPS-IVDs and LPS-treated NP/AF cells

**Fig. 4:**
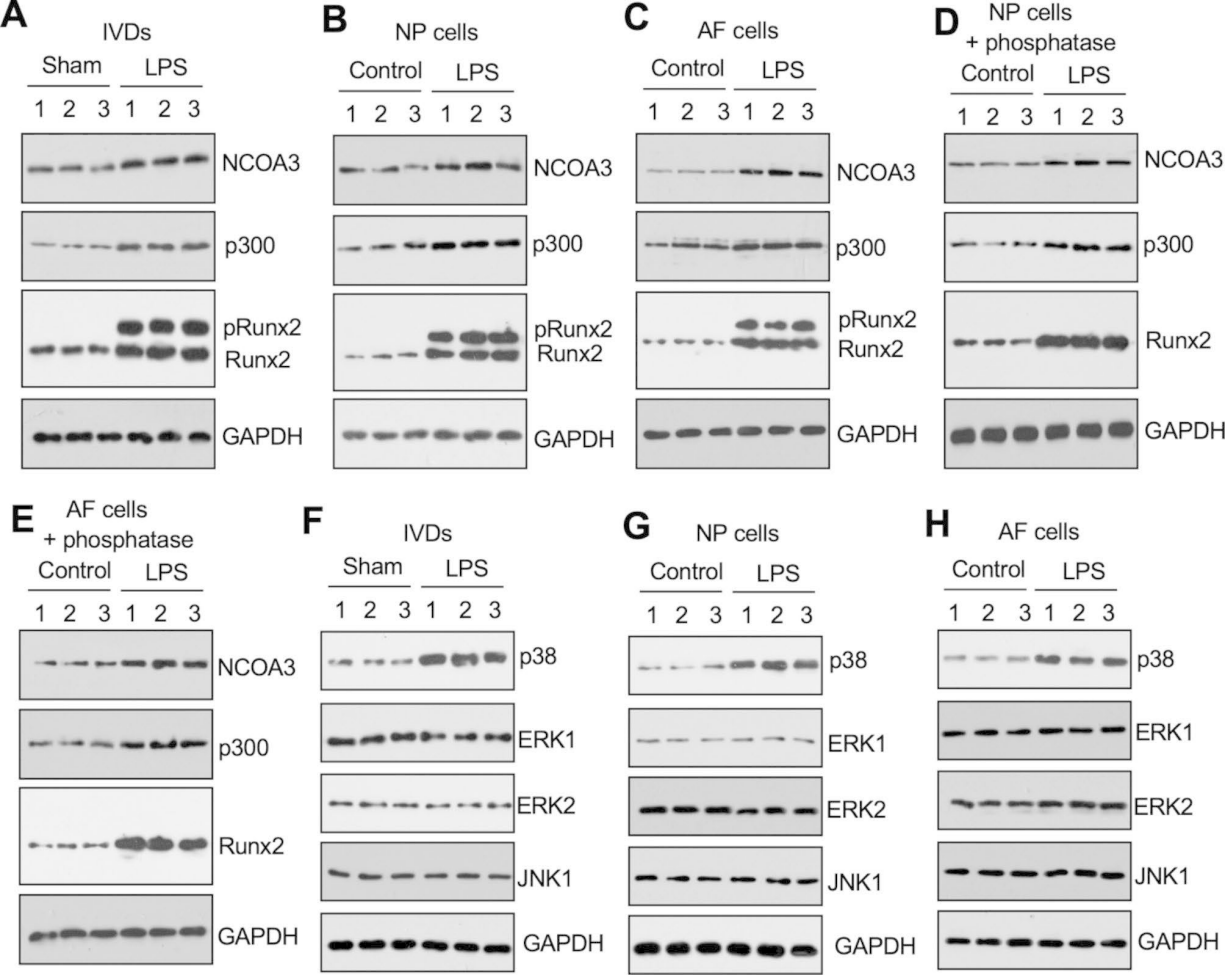
Runx2 was phosphorylated in LPS-IVDs and LPS-treated NP-1/AF-1 cells **(A)** Protein levels of NCOA3-p300-Runx2 members in IVDs from sham- and LPS-treated mice. Homogenates of three lumbar discs (L1/L2) from three sham- and LPS-treated mice were used for western blotting to detect the protein levels of NCOA3, p300, Runx2, and GAPDH (loading control). **(B** and **C)** Protein levels of NCOA3-p300-Runx2 members in LPS-treated NP and AF cells. Three NP/AF cell lines (1, 2, and 3) were incubated with or without 20 ng/mL LPS for 6 h. Cell lysates were used for western blotting to determine the protein levels of NCOA3, p300, Runx2, and GAPDH (loading control). **(B)** NP cells; **(C)** AF cells. **(D** and **E)** Protein levels of NCOA3-p300-Runx2 members in NP and AF cells co-treated with LPS and phosphatase. Three NP/AF cell lines (1, 2, and 3) were incubated with or without 20 ng/mL LPS and 200 units of phosphatase for 6 h. Cell lysates were used for western blotting to determine the protein levels of NCOA3, p300, Runx2, and GAPDH (loading control). **(D)** NP cells; **(E)** AF cells. **(F-H)** Protein levels of different kinases in LPS-treated IVDs and LPS-treated NP-1/AF-1 cells. The same protein samples as in (A-C) were used for western blotting to determine the protein levels of p38, ERK1, ERK2, JNK1, and GAPDH (loading control). **(F)** IVDs; **(G)** NP cells; **(H)** AF cells

We examined the protein levels of the NCOA3, p300, and Runx2 members of the suspected transcriptional complex in sham-IVDs and LPS-IVDs (n = 3 for each). We observed only a slight induction of both NCOA3 and p300 in the LPS-IVDs compared to the sham-IVDs (Fig. 4A). Runx2 was significantly more abundant in the LPS-IVDs (Fig. 4A). We also found an interesting band that ran above Runx2 in the LPS-IVDs but was absent from the sham-IVDs (Fig. 4A). Administering a low dose of LPS (20 ng/mL) to primary NP and AF cells for 6 h resulted in similar expression patterns of NCOA3, p300, and Runx2 (Fig. 4B C). Co-treatment of NP and AF cells with phosphatase and LPS resulted in disappearance of the larger band running above Runx2 (Fig. 4D and E). Therefore, we speculated that the larger band was the phosphorylated form of Runx2 (pRunx2).

We investigated the kinase that phosphorylated Runx2 by examining LPS-IVDs and LPS-treated NP/AF cells for the presence of several kinases, including p38, extracellular signal-regulated kinase 1 (ERK1), ERK2, and c-Jun N-terminal kinase 1 (JNK1). Of these, only p38 kinase was significantly induced in both LPS-IVDs and LPS-treated NP/AF cells (Fig. 4F H).

We then generated p38-KD cells in both NP-1 and AF-1 backgrounds (Figures S6A-S6D). These cells showed significantly decreased protein levels of pRunx2 when treated with 20 ng/mL LPS (Figures S6E and S6F). Co-treatment of NP-1 and AF-1 cells with LPS and a p38 inhibitor (AL8697 or AMG548) revealed that both p38 inhibitors dose-dependently decreased the protein level of pRunx2 (Figures S6G and S6H).

Several serine sites, including serine-28 (Ser), Ser-275, and Ser-340, have been identified on Runx2 as phosphorylation sites for kinases [21, 22]. Examination of whether any of these sites were phosphorylated in vivo in LPS-IVDs and in vitro in LPS-treated NP/AF cells revealed that Ser-28, but not Ser-275 or Ser-340, was phosphorylated following LPS treatment (Figure S7).

### pRunx2^S28^ bound to the ***ADAMTS*** promoters with a much higher affinity than Runx2

**Fig. 5:**
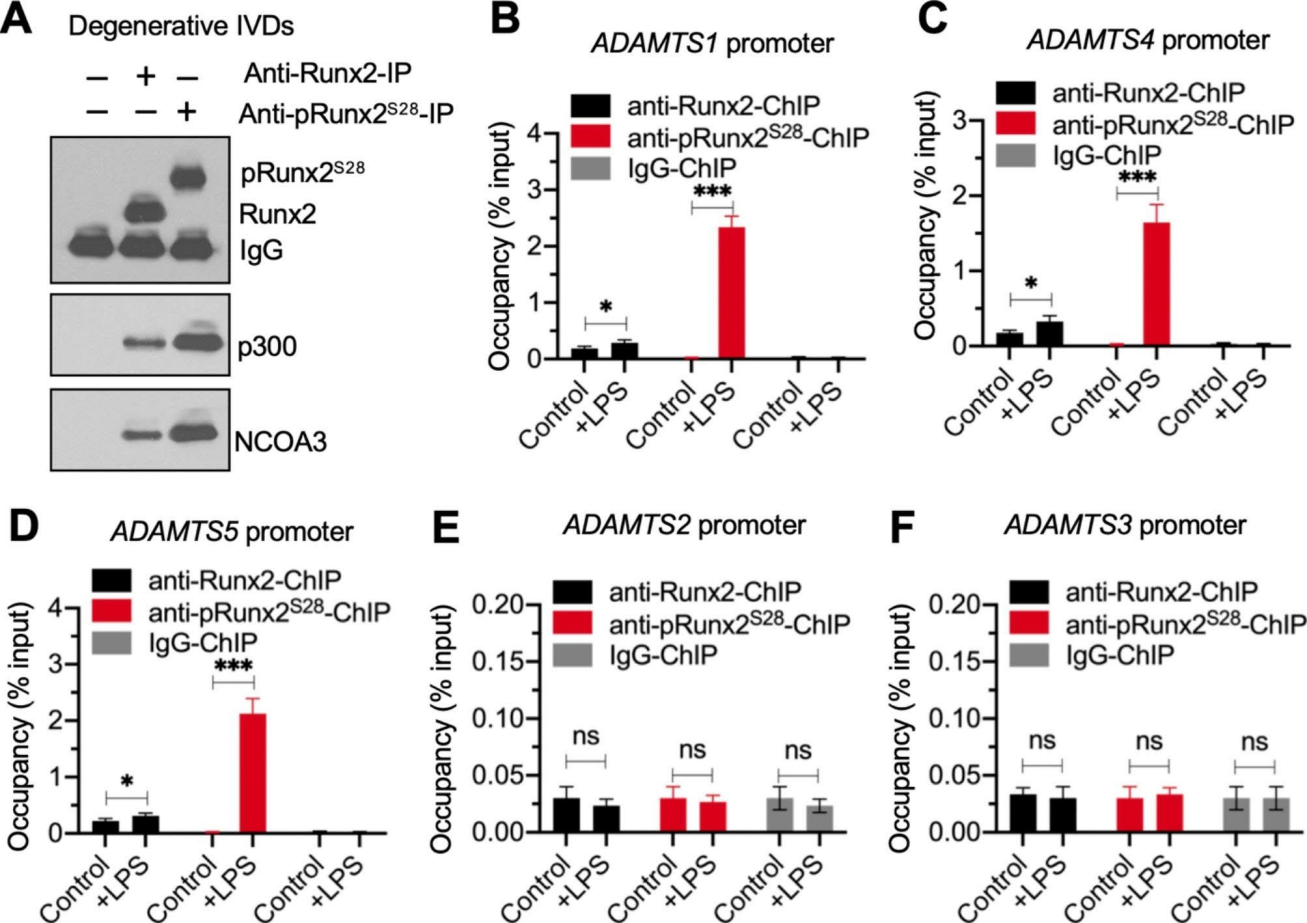
The binding affinity on the promoters of *ADAMTS* genes was much higher for pRunx2^S28^ than for Runx2 **(A)** Greater amounts of p300 and NCOA3 protein were pulled down by pRunx2 than by Runx2. Equal weights (0.05 g) of three independent IVDs from LPS-challenged mice were mixed to make a homogenate, followed by immunoprecipitation with anti-Runx2-, anti-pRunx2^S28^, and IgG-coated protein A agarose. The purified complexes were used to determine the protein levels of Runx2, pRunx2^S28^, p300, and NCOA3. **(B-F)** Occupancies of Runx2 and pRunx2^S28^ on the promoters of *ADAMTS1/2/3/4/5*. The NP-1 cells were incubated with or without 20 ng/mL LPS for 6 h, followed by ChIP assays with anti-Runx2-, anti-pRunx2^S28^, and IgG-coated protein A agarose. The input and output DNA samples were used for RT-qPCR analyses to examine the occupancies of Runx2 and pRunx2^S28^ on the promoters of *ADAMTS1***(B)**, *ADAMTS4***(C)**, *ADAMTS5***(D)**, *ADAMTS2***(E)**, and *ADAMTS3***(F)**. *P < 0.05; ***P < 0.001; ns: no significant difference

Since the expression of both total Runx2 and pRunx2 was upregulated following LPS treatment, we determined whether pRunx2 also recruited p300 and NCOA3 to form a complex. IP experiments performed with anti-Runx2-, anti-pRunx2^S28^-, and IgG-coated agarose in the same materials shown in Fig. 3A revealed a greater pulldown of p300 and NCOA3 by pRunx2^S28^ than by similar amounts of Runx2 (Fig. 5A).

ChIP RT-qPCR assays using anti-Runx2-, anti-pRunx2^S28^, and IgG-coated protein A agarose showed that Runx2 occupied the promoters of *ADAMTS1/4/5*, but not *ADAMTS2/3*, in NP-1 cells without LPS treatment (Fig. 5B F). The Runx2 occupancy on the promoters of *ADAMTS1/4/5* was only slightly increased after administering LPS (Fig. 5B and D). We did not detect pRunx2^S28^ binding on the promoters of *ADAMTS1/4/5* in the absence of LPS treatment (Fig. 5B and D), whereas the binding efficiency on the promoters of *ADAMTS1/4/5* was approximately 8-fold higher for pRunx2^S28^ than for Runx2 in LPS-treated NP-1cells (Fig. 5B and D). These results suggested that pRunx2^S28^ might play a dominant role in the assembly of a complex with p300 and NCOA3 that transactivates *ADAMTS* expression following LPS exposure.

### USP24 protected pRunx2^S28^ from ubiquitin-dependent proteasomal degradation

**Fig. 6:**
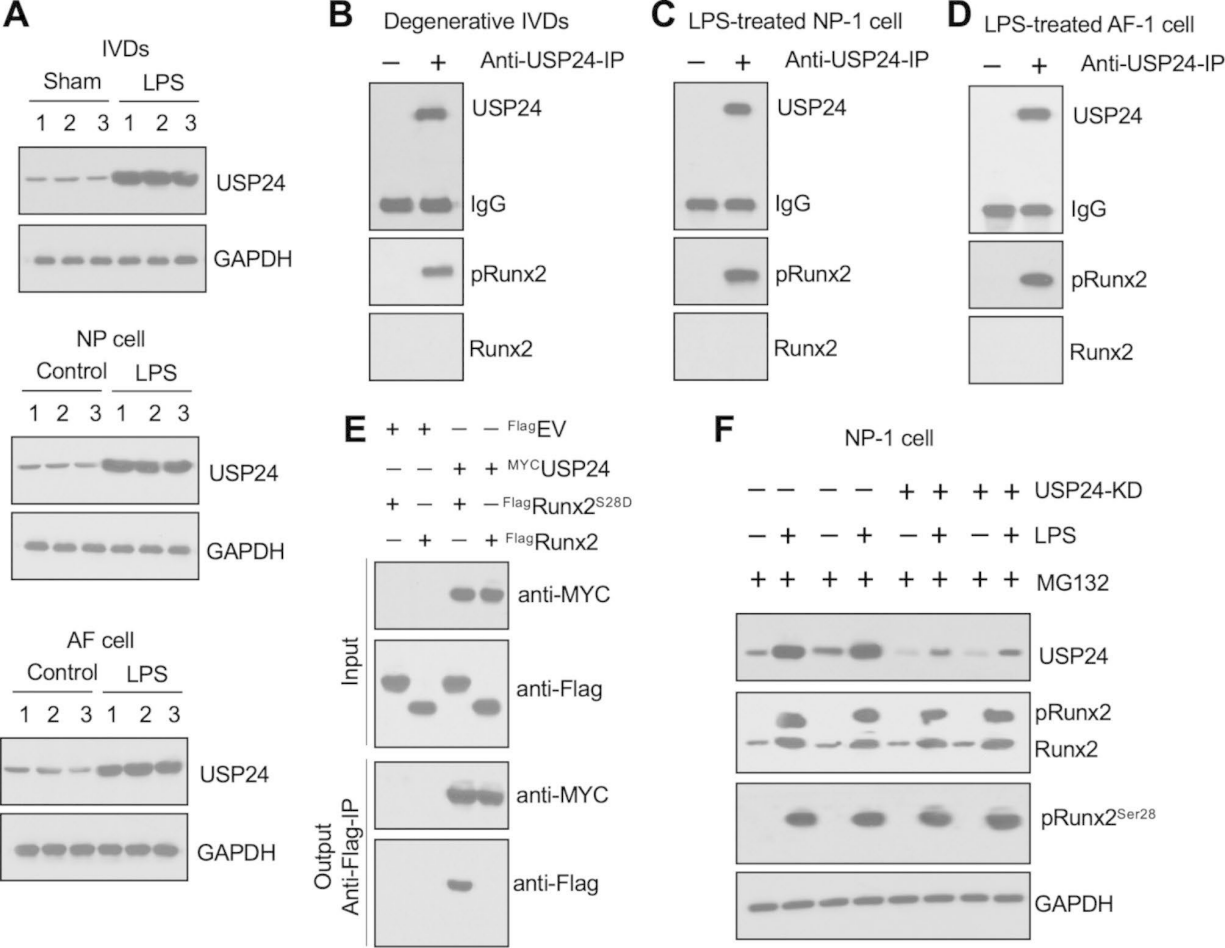
USP24 interacted with pRunx2^S28^ and protected pRunx2^S28^from proteasomal degradation **(A)** Protein levels of USP24 in LPS-treated IVDs and LPS-treated NP-1/AF-1 cells. The protein samples used to detect the USP24 protein levels were the same ones used in Fig. 4A C. **(B)** USP24 pulled down pRunx2^S28^ but not Runx2 in LPS-IVDs. Equal weights (0.05 g) of three independent IVDs from LPS-challenged mice were mixed to make a homogenate, followed by immunoprecipitation with anti-USP24-coated and IgG-coated protein A agarose. The purified complexes were used for western blotting assays with anti-USP24, anti-pRunx2^S28^, and anti-Runx2 antibodies. **(C** and **D)** USP24 pulled down pRunx2^S28^, but not Runx2, in LPS-treated NP-1/AF-1 cells. Cell lysates from LPS-treated NP-1/AF1 cells were used for immunoprecipitation with anti-USP24- and IgG-coated protein A agarose. The purified complexes were used for western blotting assays with anti-USP24, anti-pRunx2^S28^, and anti-Runx2 antibodies. **(E)** In vitro Co-IP results. The ^Myc^USP24 plasmid was cotransfected with ^Flag^Runx2 or ^Flag^pRunx2^S28D^ into NP-1 cells. After incubation at 37 °C for 48 h, the cells were used for immunoprecipitation using anti-Myc-agarose. The input and output proteins were detected with anti-Flag and anti-Myc antibodies. **(F)** MG132 blocked the degradation of pRunx2, which is dependent on USP24 depletion. The Control-KD1/2 and USP24-KD1/2 cells were co-treated with LPS and MG132 for 6 h, followed by protein isolation and western blotting to determine the protein levels of USP24, Runx2/pRunx2, pRunx2^S28^, and GAPDH

We explored the possibility that pRunx2^S28^ could recruit other proteins for the transactivation of *ADAMTS* genes by performing IP assays using anti-pRunx2^S28^-coated protein A agarose in LPS-IVDs and analyzing the pRunx2^S28^-interacting proteins by mass spectrometry. Comparison of the lists of candidate pRunx2^S28^-interacting proteins with Runx2-interacting proteins revealed a high abundance of a deubiquitinating enzyme called USP24 (ubiquitin-specific peptidase 24) amid the pRunx2-immunoprecipitated proteins (Tables S6 and S7). The protein level of USP24 was significantly increased in LPS-IVDs and LPS-treated NP-1/AF-1 cells (Fig. 6A), and USP24 immunoprecipitated pRunx2^S28^, but not Runx2, in LPS-IVDs and LPS-treated NP/AF cells (Fig. 6B and D). Mutation of the serine-28 site of Runx2 to aspartate (D) to mimic Runx2 phosphorylation revealed that USP24 directly interacted with Runx2^S28D^ rather than Runx2 in NP-1 cells co-expressing ^Flag^Runx2^S28D^+^MYC^USP24 (Fig. 6E).

We also generated USP24-KD cell lines in both NP and AF backgrounds (Figures S8A-S8D) and then treated them with 20 ng/mL LPS. Depletion of USP24 caused a significant reduction in pRunx2^S28^ (Figures S8E and S8F). Cotreatment of the USP24-KD cell lines with the proteasome inhibitor MG132 and LPS resulted in an accumulation of pRunx2^S28^ to a comparable level to that observed in LPS-treated NP/AF cells (Fig. 6F and S8G). To provide evidence that pRunx2 was a direct target of USP24, we conducted both in vivo and in vitro deubiquitination assays. In the in vivo experiments, we observed that the depletion of USP24 led to an increase in the ubiquitination of pRunx2, whereas the overexpression of USP24 resulted in a decrease in pRunx2 ubiquitination in LPS-treated NP-1 cells (Figure S9A). Furthermore, our in vitro findings demonstrated that recombinant USP24 exhibited a dose-dependent ability to cleave and remove the ubiquitin moiety from pRunx2 (Figure S9B). These results suggested that USP24 protected pRunx2 from ubiquitin-dependent proteasomal degradation following LPS treatment.

Examination of the mRNA levels of *ADAMTS1/2/3/4/5* in USP24-KD cells treated with LPS alone and co-treated with MG132 and LPS indicated that the depletion of USP24 significantly decreased the expression levels of *ADAMTS1/4/5* without changing the mRNA levels of *ADAMTS2/3* following LPS treatment (Figures S10A and S11A). The suppression of *ADAMTS1/4/5* expression in LPS-treated USP24-KD cells could be overcome by MG132, which restored *ADAMTS1/4/5* expression to the same levels observed in the LPS-treated Control-KD cells (Figures S10B and S11B).

### Inhibitors of NCOA3, p300, and p38 blocked the expression of ***ADAMTS*** genes in LPS-treated NP/AF cells

Figure 2 shows that the depletion of Runx2 caused a downregulation of 13 *ADAMTS* genes, without affecting the expression of the other 6 *ADAMTS* genes. We assumed that inhibition of either NCOA3 or p300, as well as the blockage of Runx2 phosphorylation by p38 inhibitors, would suppress the expression of the 13 *ADAMTS* genes. Treatment of NP-1/AF-1 cells with the NCOA3 inhibitor bufalin (10 nM), the p300 inhibitor EML425 (1 µM), or the p38 inhibitor doramapimod (40 nM) in the presence of 20 ng/mL LPS significantly suppressed the downregulation of *ADAMTS-1*, -*4, -5, -6*, -*7, -8*, -*9*, -*14*, -*15*, -*16*, -*17*, -*18*, and − *20* but did not affect the expression of *ADAMTS*-*2, -3*, -*10*, -*12*, -*13*, and − *19* (Figures S12 and S13).

ChIP RT-qPCR assays of p300/NCOA3/p38 inhibitor-treated cells, performed using anti-pRunx2^S28^-, anti-p300-, anti-NCOA3-, and IgG-coated protein A agarose, with or without incubation with 20 ng/mL LPS, revealed decreased occupancies of NCOA3-p300-pRunx2^S28^ members on the promoters of *ADAMTS1/4/5*, whereas the NCOA3-p300-pRunx2^S28^ members did not bind to the promoters of *ADAMTS2/3* (Figures S14 and S15). Treatment with LPS alone increased the occupancies of all NCOA3-p300-pRunx2^S28^ members on the promoters of *ADAMTS1/4/5*, but this induction was significantly reduced by treatment with the p300, NCOA3, or p38 inhibitors (Figures S14 and S15).

### Administration of NCOA3, p300, and p38 inhibitors prevented lumbar disc degeneration in LPS-treated mice

**Fig. 7:**
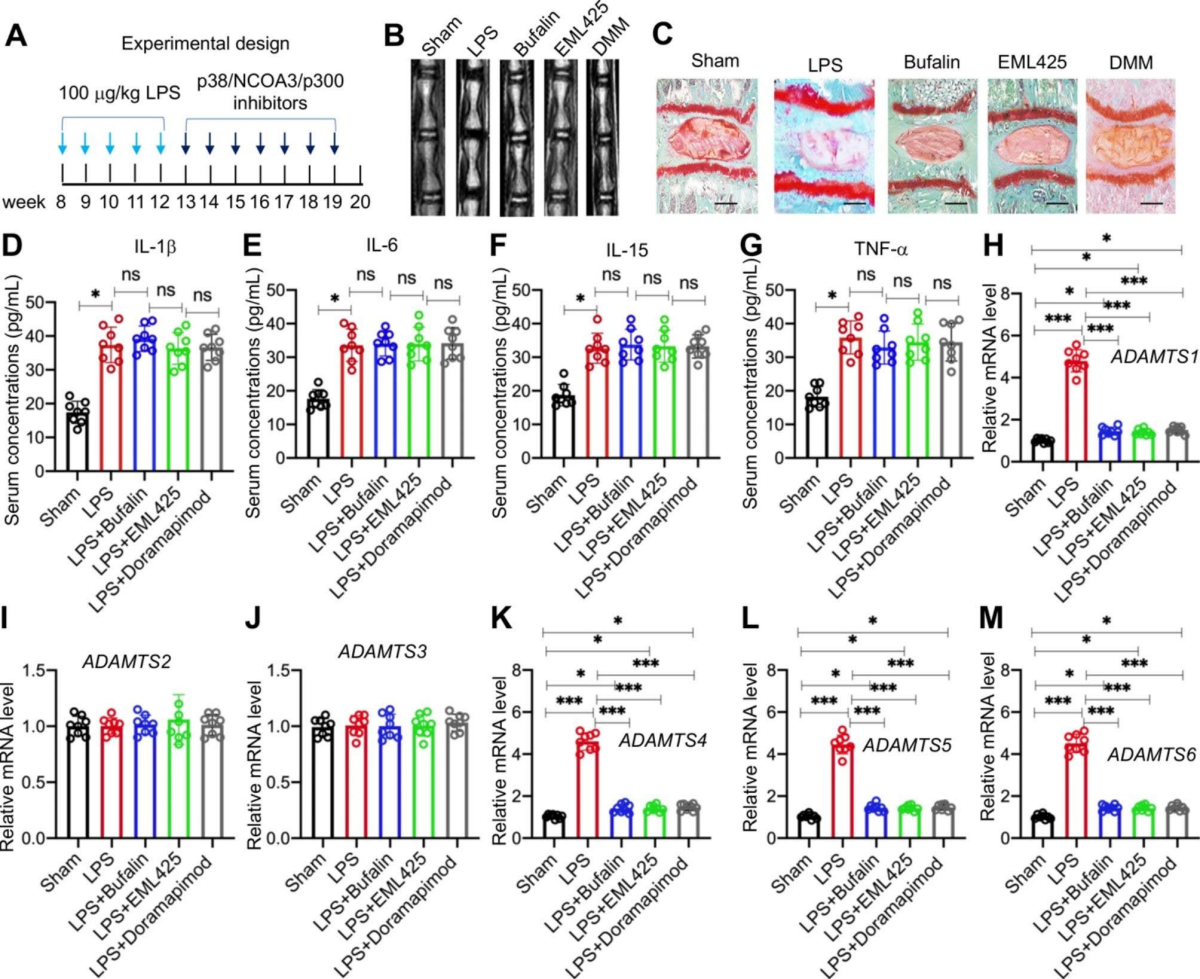
Inhibitors of p38/NCOA3/p300 decreased the expression of *ADAMTS* genes and slowed the degeneration of IVDs in LPS-challenged mice **(A)** The injection time points for LPS and p38/NCOA3/p300 inhibitors. **(B)** Representative MRI images of lumbar IVDs from different groups of mice. **(C)** Representative picrosirius red staining images of IVDs from different groups of mice. Bars = 100 μm. **(D-G)** Serum concentrations of proinflammatory cytokines: IL-1β **(D)**, IL-6 **(E)**, IL-15 **(F)**, and TNFα **(G). (H-M)** The mRNA expression levels of *ADAMTS* genes in different groups of mice (n = 8 for each group). **(H)***ADAMTS1*; **(I)***ADAMTS2*; **(J)***ADAMTS3*; **(K)***ADAMTS4*; **(L)***ADAMTS5*; **(M)***ADAMTS6*; *P < 0.05; **P < 0.01; ***P < 0.001; ns: no significant difference

The effects of NCOA3, p300, and p38 inhibitors were also investigated in vivo by pretreating mice with 100 µg/kg LPS for 4 weeks and then treating them with inhibitors weekly for a further 8 weeks (Fig. 7A). MRI images and histological results showed that lumbar IVDs in bufalin-, EML425-, and doramapimod-treated mice showed no obvious degeneration, while the lumbar IVDs in the mice only challenged with LPS (sham group) showed significant degenerative changes (Fig. 7B C).

Measurements of circulating concentrations of proinflammatory cytokines revealed that the administration of NCOA3, p300, and p38 inhibitors did not change the serum concentrations of IL-1β, IL-6, IL-15, or TNF-α (Fig. 7D and G). Similar to the in vitro results, treatments with bufalin, EML425, or doramapimod markedly decreased the expression levels of the 13 downregulated *ADAMTS* genes in lumbar IVDs (Fig. 7H M, and S16). These results suggested that the inhibition of the NCOA3-p300-pRunx2 complex could prevent disc degeneration in mice with chronic inflammation.

## Discussion

**Fig. 8:**
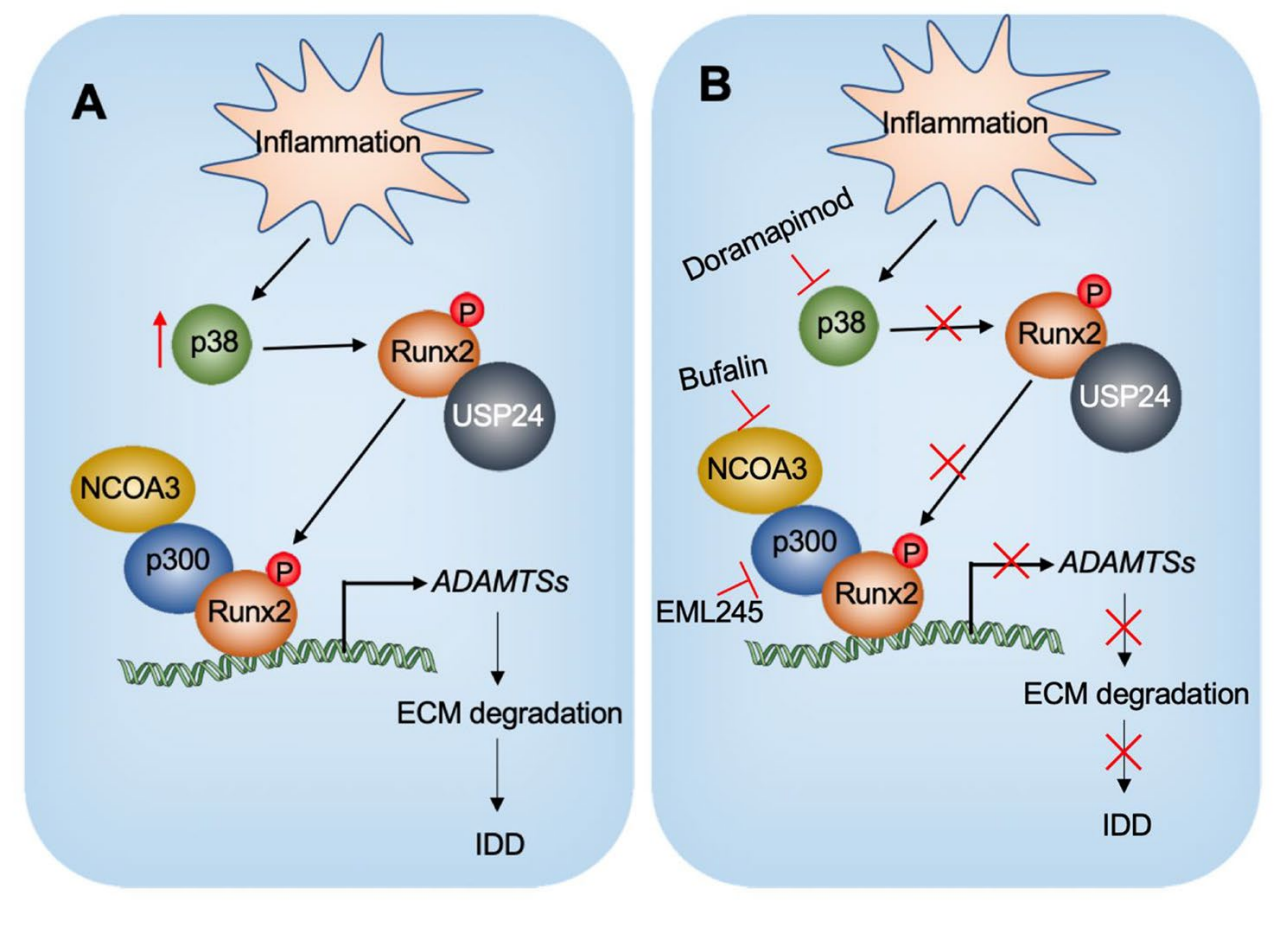
Schematic model for the degeneration of IVDs by the transactivation of *ADAMTS* genes by the NCOA3-p300-pRunx2 complex **(A)** Schematic model of the transactivation of *ADAMTS* genes by the NCOA3-p300-pRunx2 complex in the degeneration of IVDs. Chronic inflammation activates p38 kinase, which phosphorylates Runx2 at the Ser28 site. pRunx2^S28^ then recruits p300 and NCOA3 to assemble a complex that binds to the promoters of *ADAMTS1/4/5/6/7/8/9/10/12/13/14/15/16/17/18/20* to transactivate their expression. The induction of these *ADAMTS* genes promotes ECM degradation and leads to IDD. **(B)** Schematic model depicting how inhibition of the NCOA3-p300-pRunx2 complex decreases the degeneration of IVDs. Administration of p38/NCOA3/p300 inhibitors in LPS-challenged mice decreases the expression of *ADAMTS1/4/5/6/7/8/9/10/12/13/14/15/16/17/18/20*, thereby retarding ECM degradation and slowing the IDD process

Chronic inflammation is prevalent in patients with IDD, but the causal relationship between chronic inflammation and IDD remains controversial [1, 17]. In this study, we provide evidence that chronic inflammation induces *ADAMTS* genes and promotes IDD occurrence through a mechanism involving an NCOA3-p300-pRunx2^S28^ complex. The chronic inflammation microenvironment in LPS-challenged mice activates p38 kinase, which phosphorylates Runx2, enabling it to recruit USP24 for stabilization. The stabilized phosphorylated Runx2 then assembles a transcriptional complex with p300 and NCOA3 to transactivate 13 *ADAMTS* genes whose promoters contain the Runx2 binding sites. Overexpression of the *ADAMTS* genes promotes ECM degradation and induces IDD (Fig. 8A). Inhibitors of NCOA3, P300, and p38 can block the function of the NCOA3-p300- pRunx2^S28^ complex, thereby inhibiting the expression of the 13 *ADAMTS* genes, decreasing ECM degradation, and preventing IDD (Fig. 8B). Our study not only reveals the causal relationship between chronic inflammation and IDD, but it also identifies a therapeutic strategy for the treatment of IDD caused by chronic inflammation.

Although some *ADAMTS* genes have shown elevated expression in degenerative IVDs [14], little is known about their transcriptional activation. Some publications have reported that proinflammatory cytokines (e.g., IL-1β, IL6, and TNF-α) can induce the expression of *ADAMTS4*, -*5*, and − *8* in different cell types, including chondrocytes, cardiomyocytes, and NP cells [23–25]. *ADAMTS1* can also be regulated by progesterone and luteinizing hormones during ovulation [26]. However, the expression patterns of all *ADAMTS* genes modulated during the IDD pathological process are not yet established. In this study, we explored the expression levels of all 19 *ADAMTS* genes expressed in degenerative IVDs from mice challenged with LPS. A majority of these *ADAMTS* genes were induced by the NCOA3-P300-pRunx2^S28^ complex in degenerative IVDs. To our knowledge, this study is the first to elucidate the expression patterns and regulatory mechanisms controlling *ADAMTS* gene expression under chronic inflammatory conditions.

The transcriptional regulation of Runx2 itself is weak; consequently, it needs to recruit different transcriptional regulatory proteins, such as TAZ (transcriptional coactivator with PDZ-binding motif), pRB (retinoblastoma protein), SATB2 (Special AT-Rich Sequence-Binding Protein 2), and p300, to jointly regulate the expression of target genes [27–30]. Fine-tuning the Runx2 activity and stability is therefore crucial for the regulation of its target genes [27–30]. Some studies have shown that phosphorylation, acetylation, or ubiquitination can mediate the activity and stability of Runx2 [27–30]. In the present study, we revealed a new mechanism by which Runx2 is stabilized by p38-dependent phosphorylation and USP24 deubiquitination. Recently, Kim and colleagues found a similar mechanism for Runx2 stabilization in the process of bone formation [30]. They demonstrated that casein kinase 2 (CK2) phosphorylated RUNX2 during osteoblast differentiation and that pRunx2 recruited the deubiquitinase herpesvirus-associated ubiquitin-specific protease (HAUSP) [30]. HAUSP interacted with pRUNX2 and diverted it away from ubiquitin-dependent proteasomal degradation [30]. These results suggest that the regulation of Runx2 activity and stability is very complex, and that it may vary greatly in different biological processes.

We showed that inhibitors of NCOA3, p300, and p38 significantly suppress the expression of *ADAMTS* genes whose promoters contain the Runx2 binding sites and consequently retarded the degeneration of IVDs. Nevertheless, the specificity of the inhibitory effects on *ADAMTS* genes remains unclear, because the blockage of NCOA3, p300, or p38 may affect the expression of many genes other than *ADAMTS* genes. Much work is still needed to evaluate the effects of these inhibitors on the expression of other genes in NP/AF cells and to study the possible toxicity effects of the inhibitors after injection in mice. Nevertheless, screening inhibitors that target p300-Runx2^S28D^ and Runx2^S28D^-USP24 interactions appears to be a promising strategy for specifically decreasing *ADAMTS* expression.

## Conclusion

In summary, we showed that an inflammatory environment induced the expression of 13 of 19 *ADAMTS* genes. This enhanced expression was mediated by an NCOA3-p300-pRunx2^S28^ complex that forms after phosphorylation of Runx2 by p38 kinase. The pRunx2^S28^ in turn is stabilized by USP24. In vivo disruption of the NCOA3-p300-pRunx2^S28^ complex by NCOA3 and p300 inhibitors or by inhibition of p38 could decrease *ADAMTS* expression and retard the IDD process.

## Electronic supplementary material

Below is the link to the electronic supplementary material.

Additional File 1: Supplementary Figures and Tables

## Data Availability

The datasets used and/or analyzed during the present study are available from the corresponding author on reasonable request.
