# Supplementary material for: USP24-dependent stabilization of Runx2 recruits a p300/NCOA3 complex to transactivate ADAMTS genes and promote degeneration of intervertebral disc in chronic inflammation mice
Source: Biol Direct. 2023 Jul 6;18:37. doi: 10.1186/s13062-023-00395-5 (PMC10324278; doi:10.1186/s13062-023-00395-5)
Supplement: Supplementary file 1 — Additional File 1: Supplementary Figures and Tables [file 13062_2023_395_MOESM1_ESM.docx]

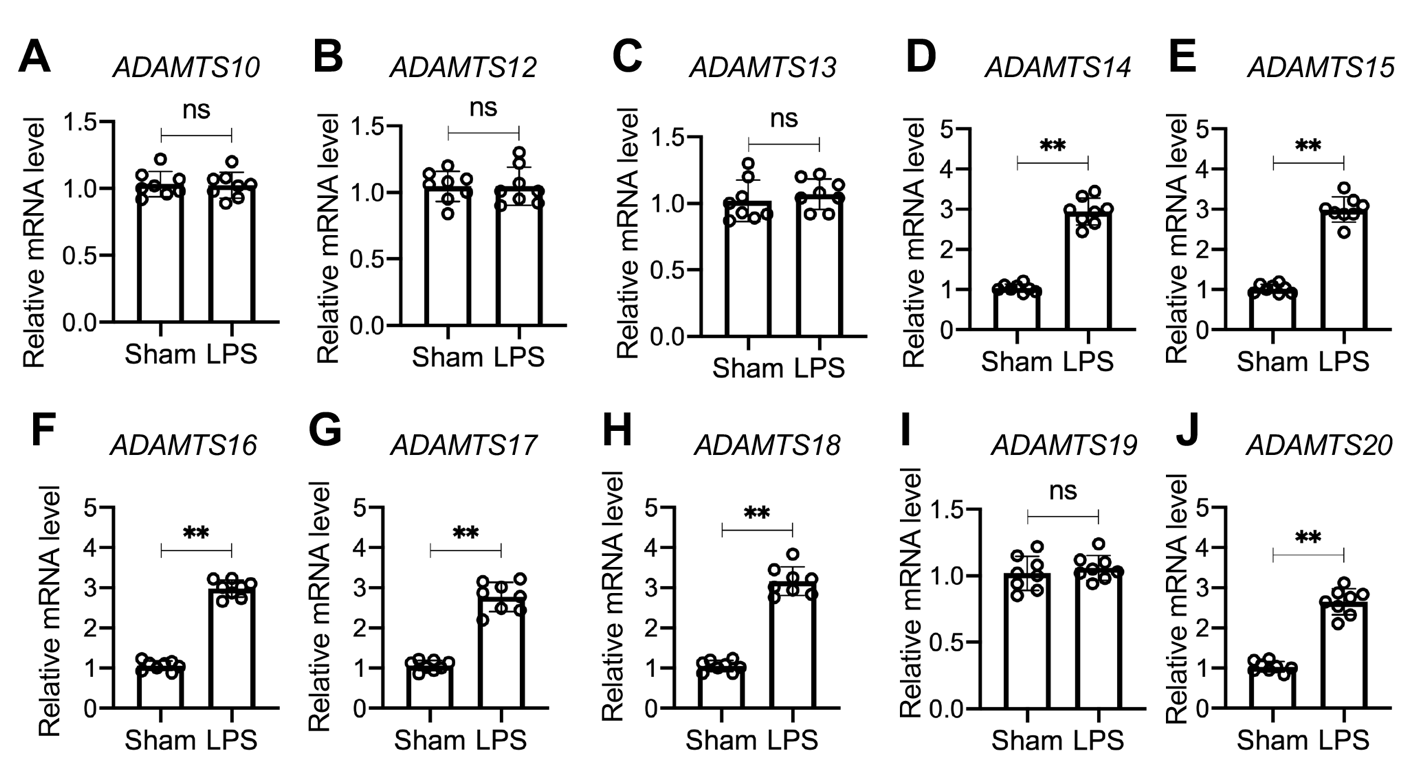


**Figure S1. The mRNA expression levels of *ADAMTSs* in IVDs from sham- and LPS-mice**

The same cDNA samples as shown in Figure 1F were used to detect mRNA levels of *ADAMTSs*. **(A)** *ADAMTS10*; **(B)** *ADAMTS12*; **(C)** *ADAMTS13*; **(D)** *ADAMTS14*; **(E)** *ADAMTS15*; **(F)** *ADAMTS16*; **(G)** *ADAMTS17*; **(H)** *ADAMTS18*; **(I)** *ADAMTS19*; **(J)** *ADAMTS20*; ***P* < 0.01; ns: no significant difference.

**
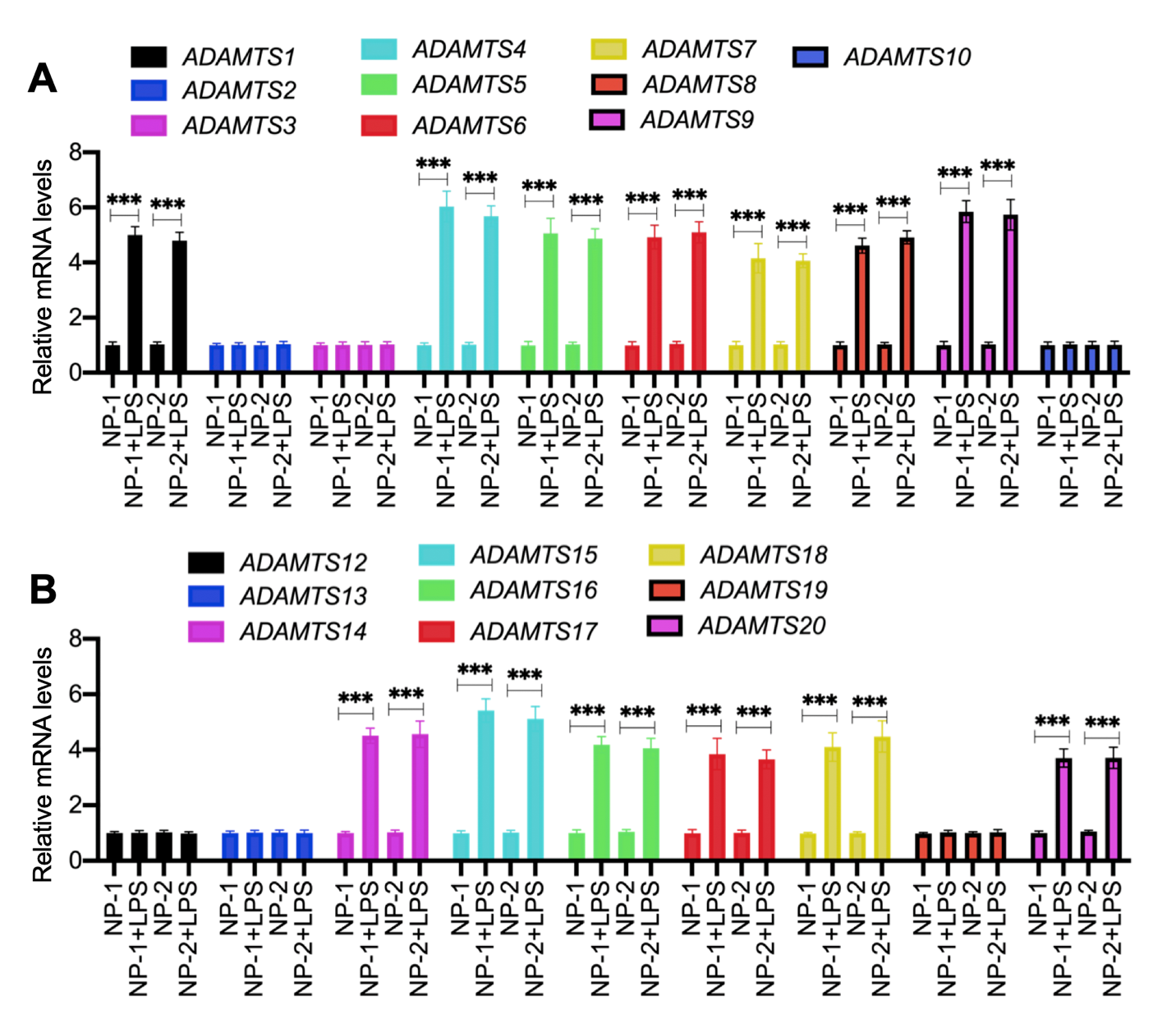
**

**Figure S2. The mRNA expression levels of *ADAMTSs* in LPS-treated NP-1/2 cells**

Two primary NP cell lines (NP-1 and -2) were treated with or without 20 ng/mL LPS for 6 h, followed by RNA isolation and RT-qPCR analyses to examine mRNA levels of *ADAMTSs*. **(A)** *ADAMTS1*, *-2*, *-3*, *-4*, *-5*, *-6*, *-7*, *-8*, *-9*, and *-10*. **(B)** *ADAMTS12*, *-13*, *-14*, *-15*, *-16*, *-17*, *-18*, *-19*, and *-20*. ****P* < 0.001.

**
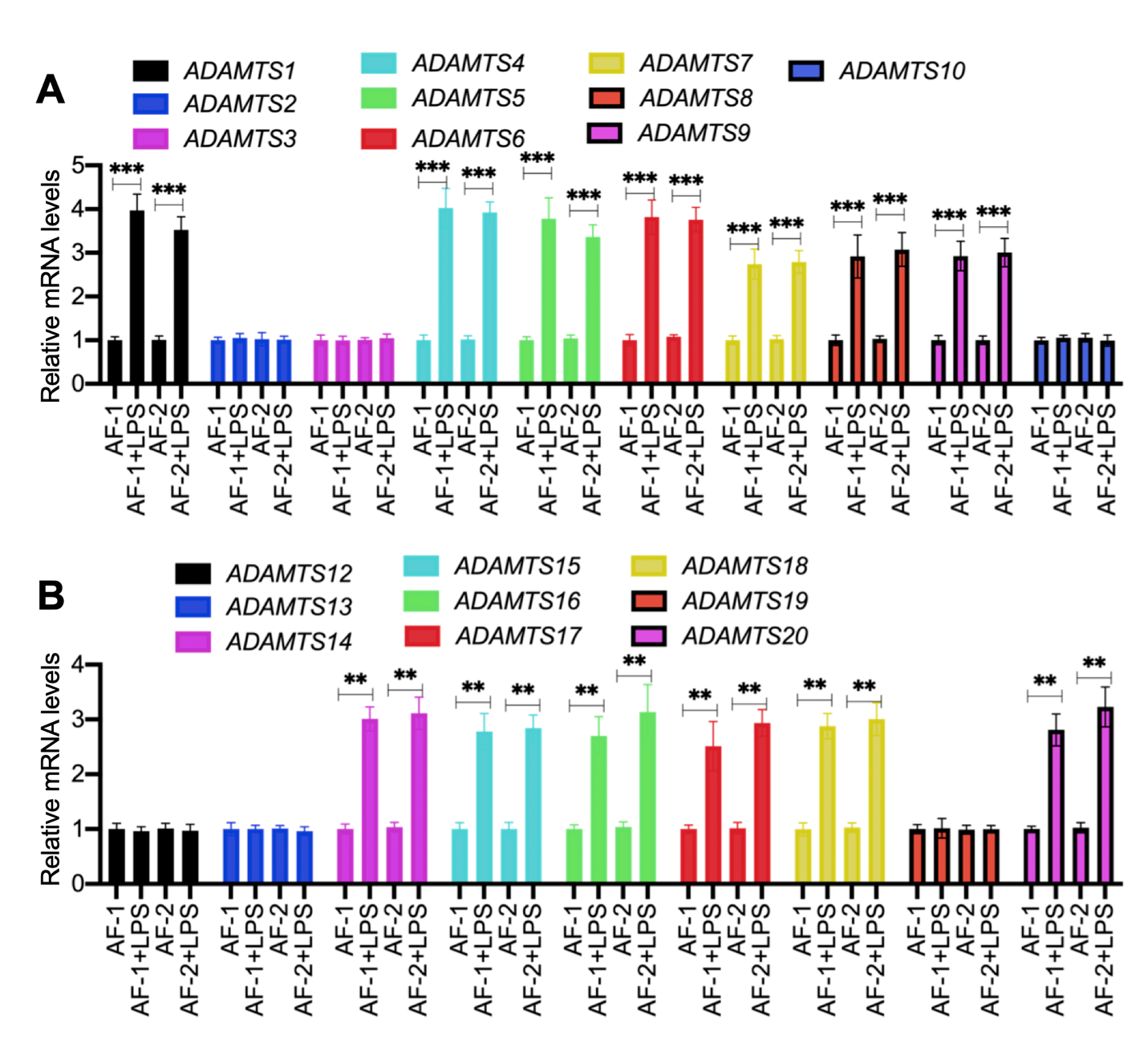
**

**Figure S3. The mRNA expression levels of *ADAMTSs* in LPS-treated AF-1/2 cells**

Two primary AF cell lines (AF1- and -2) were treated with or without 20 ng/mL LPS for 6 h, followed by RNA isolation and RT-qPCR analyses to examine mRNA levels of *ADAMTSs*. **(A)** *ADAMTS1*, *-2*, *-3*, *-4*, *-5*, *-6*, *-7*, *-8*, *-9*, and *-10*. **(B)** *ADAMTS12*, *-13*, *-14*, *-15*, *-16*, *-17*, *-18*, *-19*, and *-20*. ***P* < 0.01; ****P* < 0.001.

**
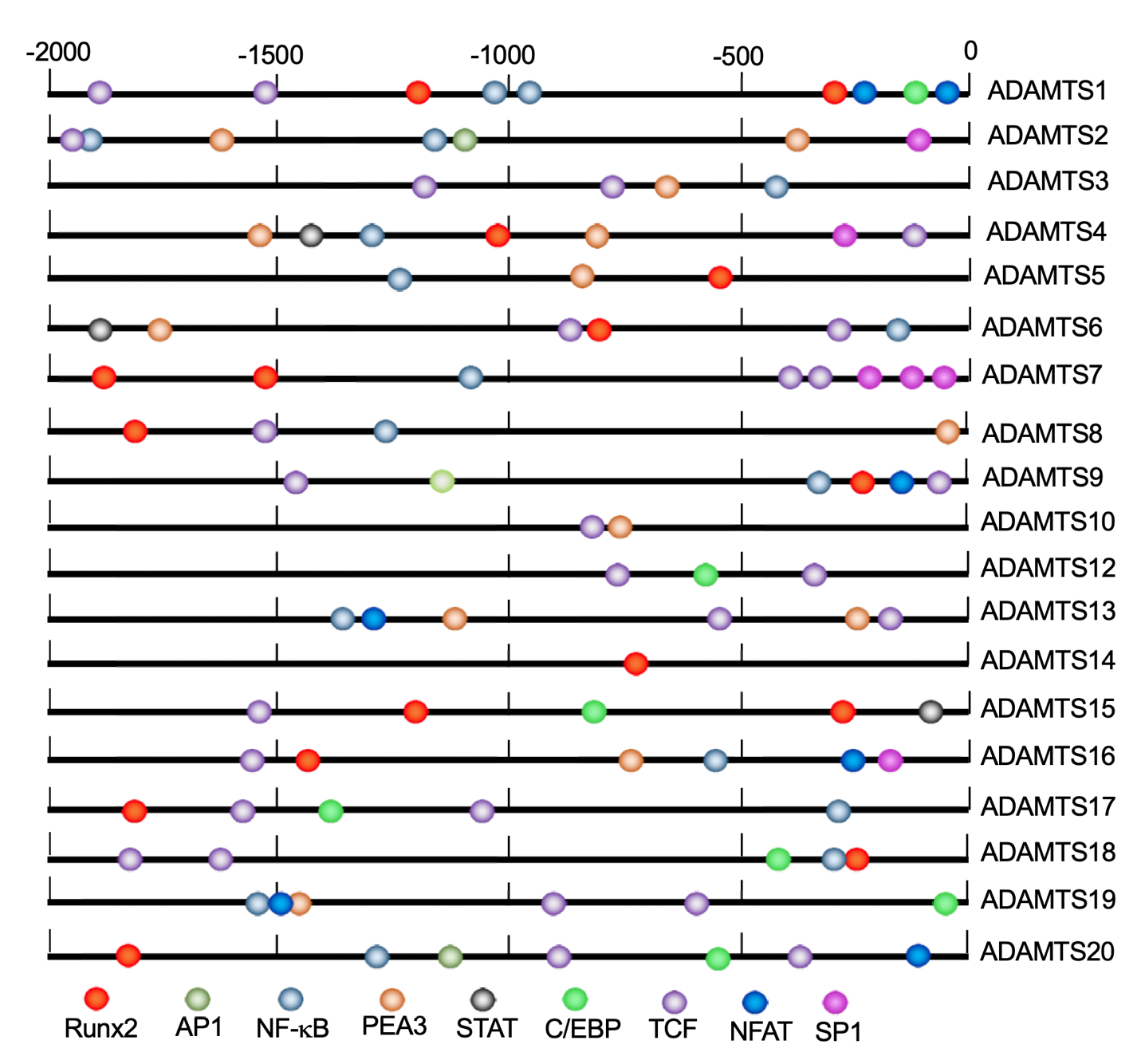
**

**Figure S4. Potential transcription factor binding sites in the promoters of mouse *ADAMTSs***

The promoters (2 kb) of 19 mouse *ADAMTS* genes were predicted the potential transcription factor binding sites on the website of <https://alggen.lsi.upc.es>. Different transcription factor binding sites were shown with different color circles.

**
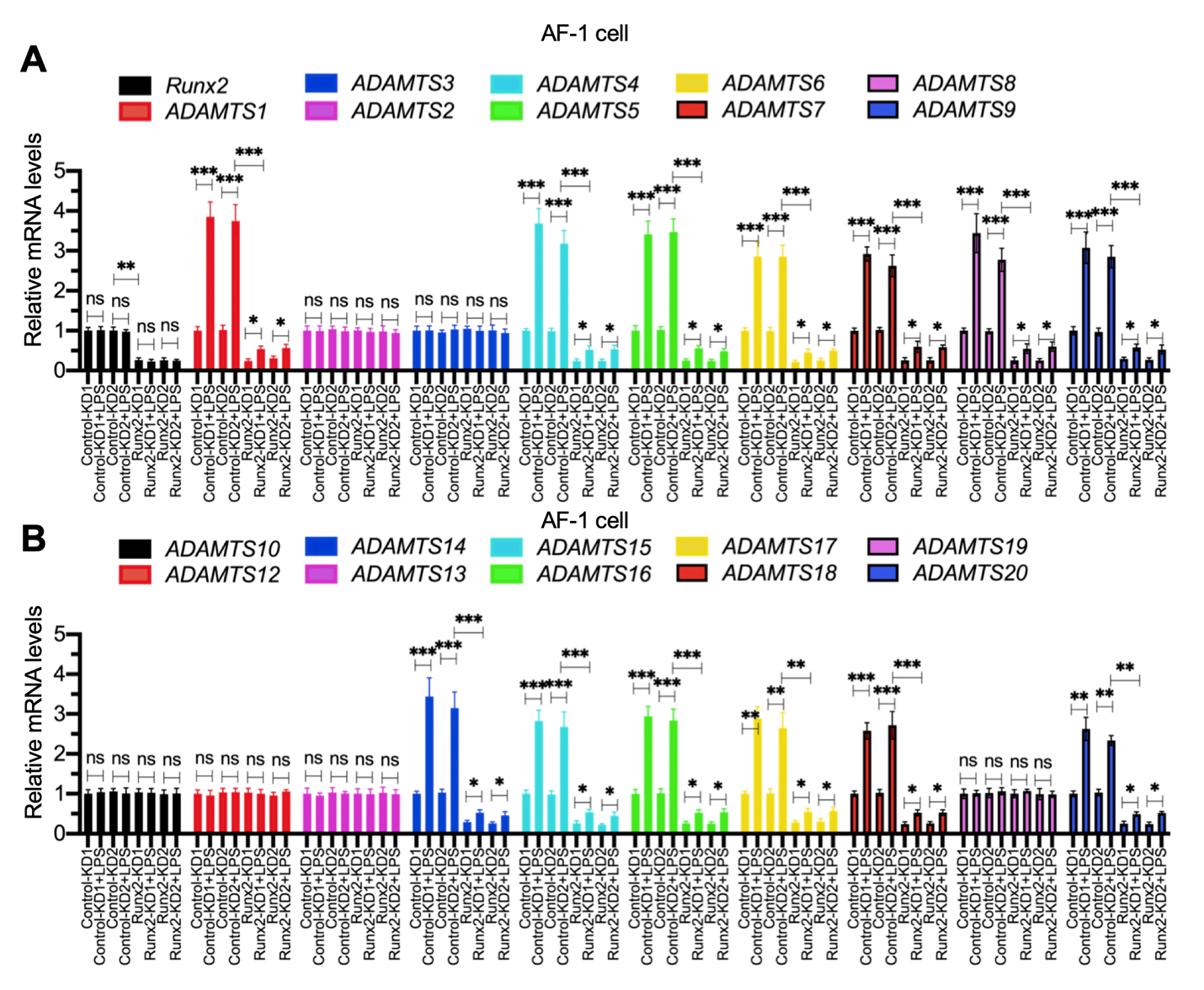
**

**Figure S5. The effects of Runx2 depletion on the expression of *ADAMTSs* in AF-1 cells treated with or without LPS**

The Control-KD1/2 and Runx2-KD1/2 cells in AF-1 background were treated with or without 20 ng/mL LPS for 6 h, followed by RNA isolation and RT-qPCR analyses to examine mRNA levels of *ADAMTSs*. **(A)** *Runx2*, *ADAMTS1*, *-2*, *-3*, *-4*, *-5*, *-6*, *-7*, *-8*, and *-9*. **(B)** *ADAMTS10*, -*12*, *-13*, *-14*, *-15*, *-16*, *-17*, *-18*, *-19*, and *-20*. **P* < 0.05; ***P* < 0.01; ****P* < 0.001; ns: no significant difference.

**
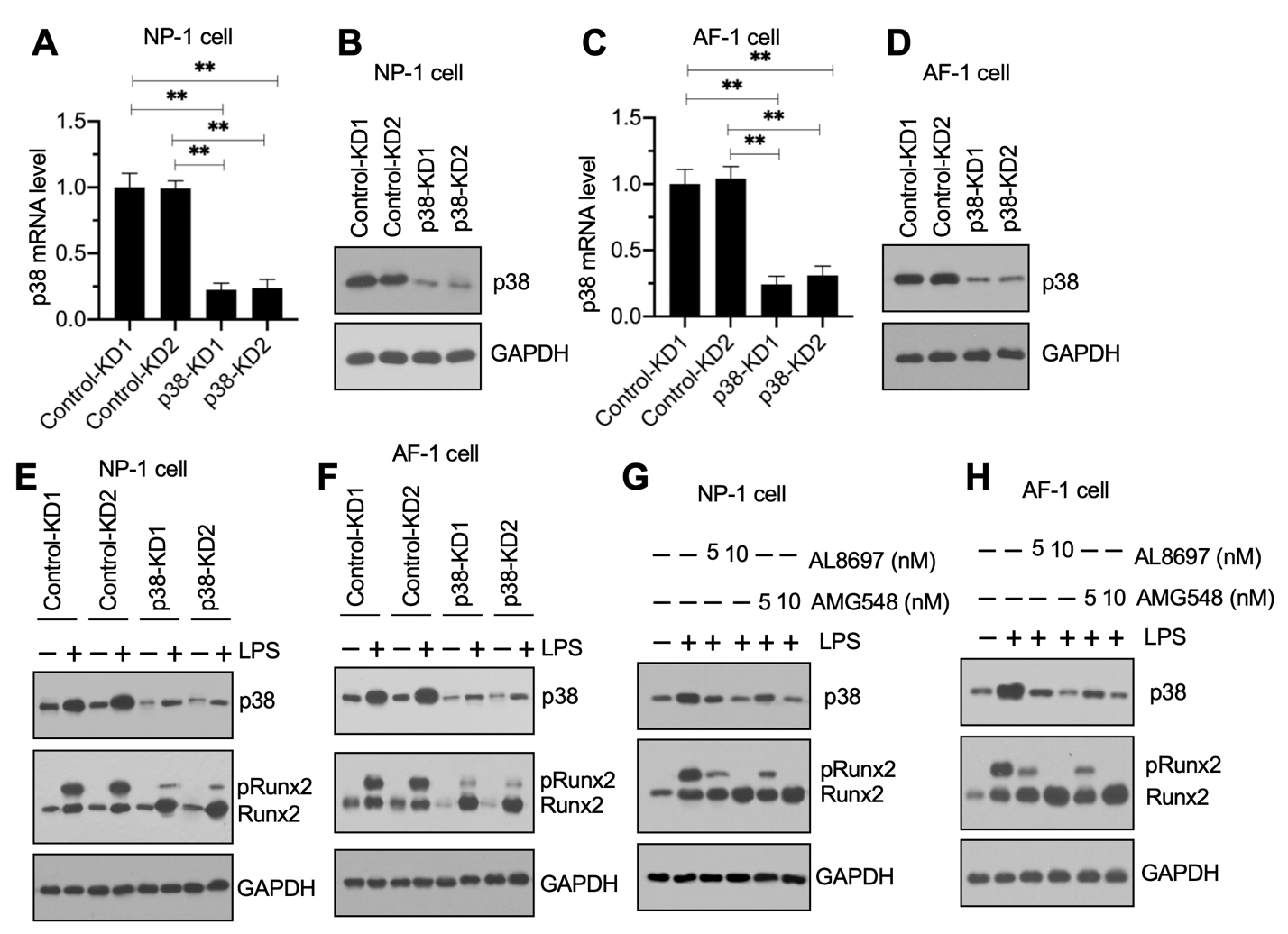
**

**Figure S6. Knockdown or inhibition of p38 decreased the phosphorylation of Runx2**

**(A)** p38 mRNA level in p38-KD1/2 cells under NP-1 background. RNA samples from Control-KD1/2 and p38-KD1/2 cells in NP-1 background were used for RT-qPCR analysis to examine the mRNA level of p38. **(B)** p38 protein level in p38-KD1/2 cells under NP-1 background. Total cell extracts from Control-KD1/2 and p38-KD1/2 cells in NP-1 background were used for immunoblots to examine protein levels of p38 and GAPDH (loading control). **(C)** p38 mRNA level in p38-KD1/2 cells under AF-1 background. RNA samples from Control-KD1/2 and p38-KD1/2 cells in AF-1 background were used for RT-qPCR analysis to examine the mRNA level of p38. **(D)** p38 protein level in p38-KD1/2 cells under AF-1 background. Total cell extracts from Control-KD1/2 and p38-KD1/2 cells in AF-1 background were used for immunoblots to examine protein levels of p38 and GAPDH (loading control). **(E** and **F)** Protein levels of p38 and Runx2/pRunx2 in p38-KD1/2 cells treated with or without LPS. The Control-KD1/2 and p38-KD1/2 cells in NP-1 background **(E)** and AF-1 background **(F)** were treated with or without 20 ng/mL LPS for 6 h, followed by protein isolation and western blotting to examine protein levels of p38, Runx2/pRunx2, and GAPDH (loading control). **(G** and **H)** Protein levels of p38 and Runx2/pRunx2 in NP-1/AF-1 cells co-treated with LPS and p38 inhibitors. The NP-1 **(G)** and AF-1 **(H)** cells were co-treated with AL8697 (5 and 10 nM) and 20 ng/mL LPS or AMG548 (5 and 10 nM) and 20 ng/mL LPS for 6 h, followed by protein isolation and western blotting to examine protein levels of p38, Runx2/pRunx2, and GAPDH (loading control). ***P* < 0.01.

**
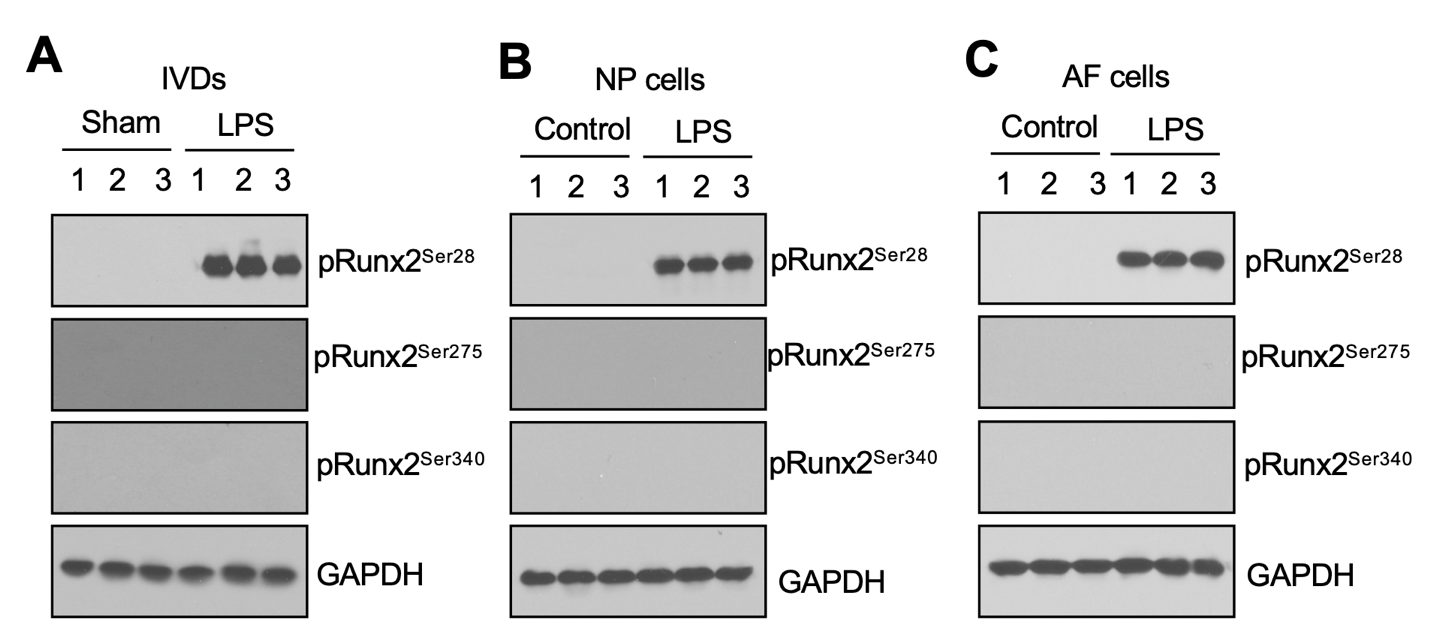
**

**Figure S7. Runx2 was phosphorylated at the Ser28 site in LPS-IVDs and LPS-treated NP-1/AF-1 cells**

**(A)** Protein levels of pRunx2^S28^, pRunx2^S275^, and pRunx2^S340^ in IVDs from sham- and LPS-mice. Homogenates of three lumbar discs (L1/L2) from three sham- and LPS-mice were used for western blotting to detect the protein levels of pRunx2^S28^, pRunx2^S275^, and pRunx2^S340^, and GAPDH. **(B** and **C)** Protein levels of pRunx2^S28^, pRunx2^S275^, and pRunx2^S340^ in LPS-treated NP **(B)** and AF **(C)** cells. Three NP/AF cell lines (1, 2, and 3) were treated with or without 20 ng/mL LPS for 6 h. Cell lysates were used for western blotting to detect the protein levels of pRunx2^S28^, pRunx2^S275^, and pRunx2^S340^, and GAPDH.

**
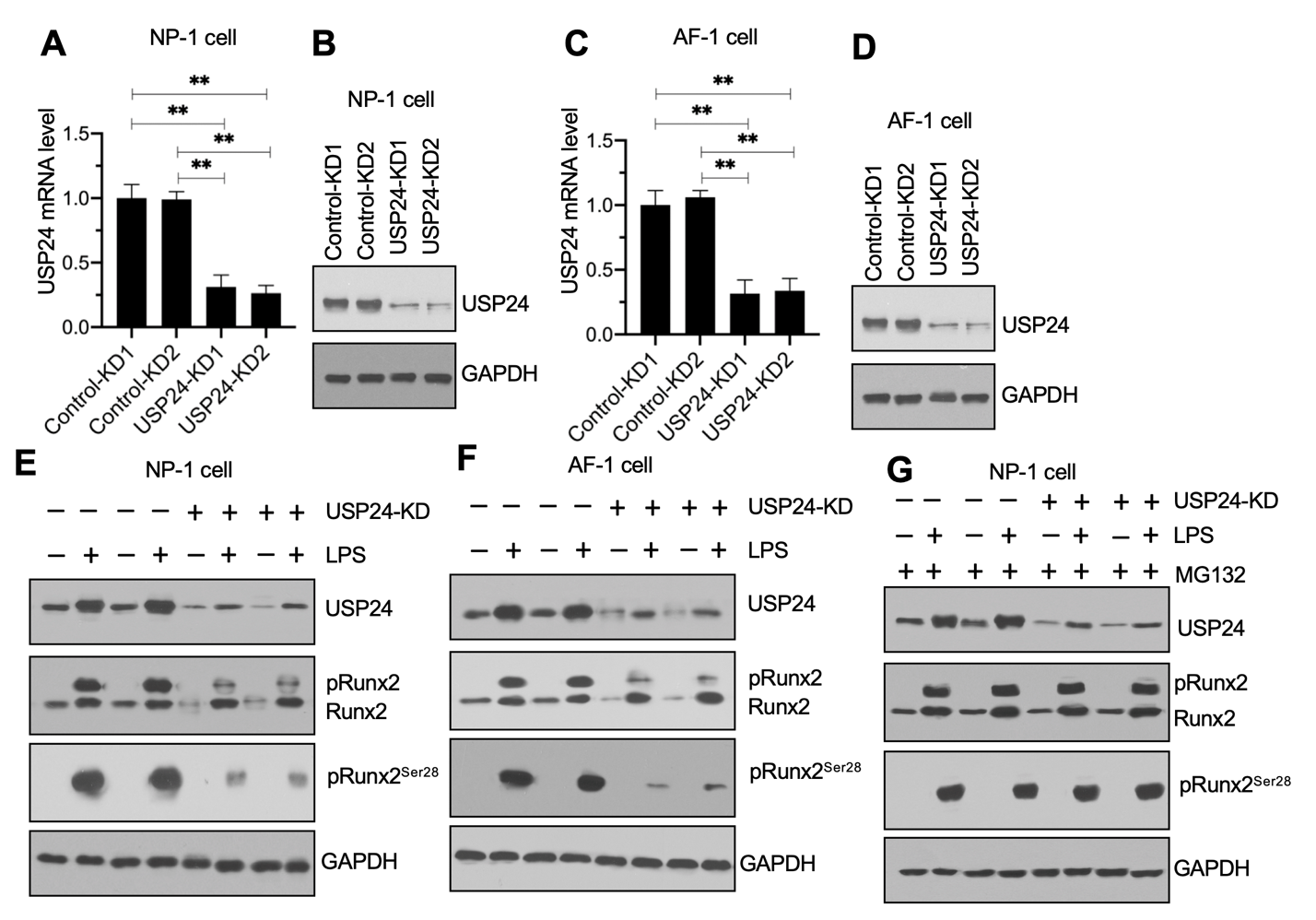
**

**Figure S8. The effects of USP24 depletion and MG132 on the protein levels of Runx2 and pRunx2^S28^**

**(A)** The *USP24* mRNA level in USP24-KD1/2 cells under NP-1 background. RNA samples from Control-KD1/2 and USP24-KD1/2 cells in NP-1 background were used for RT-qPCR analysis to examine the mRNA level of *USP24*. **(B)** The USP24 protein level in USP24-KD1/2 cells under NP-1 background. Total cell extracts from Control-KD1/2 and USP24-KD1/2 cells in NP-1 background were used for immunoblots to examine protein levels of USP24 and GAPDH (loading control). **(C)** The *USP24* mRNA level in USP24-KD1/2 cells under AF-1 background. RNA samples from Control-KD1/2 and USP24-KD1/2 cells in AF-1 background were used for RT-qPCR analysis to examine the mRNA level of *USP24*. **(D)** The USP24 protein level in USP24-KD1/2 cells under AF-1 background. Total cell extracts from Control-KD1/2 and USP24-KD1/2 cells in AF-1 background were used for immunoblots to examine protein levels of USP24 and GAPDH (loading control). **(E** and **F)** Protein levels of Runx2 and pRunx2^S28^ in USP24-KD cells treated with or without LPS. The Control-KD1/2 and USP24-KD1/2 cells in NP-1 **(E)** and AF-1 **(F)** backgrounds were co-treated with LPS and MG132 for 6 h, followed by protein isolation and western blotting to examine protein levels of USP24, Runx2/pRunx2, pRunx2^S28^, and GAPDH. **(G)** MG132 blocked the degradation of pRunx2 dependent on USP24 depletion. The Control-KD1/2 and USP24-KD1/2 cells in AF-1 background were co-treated with LPS and MG132 for 6 h, followed by protein isolation and western blotting to examine protein levels of USP24, Runx2/pRunx2, pRunx2^S28^, and GAPDH.


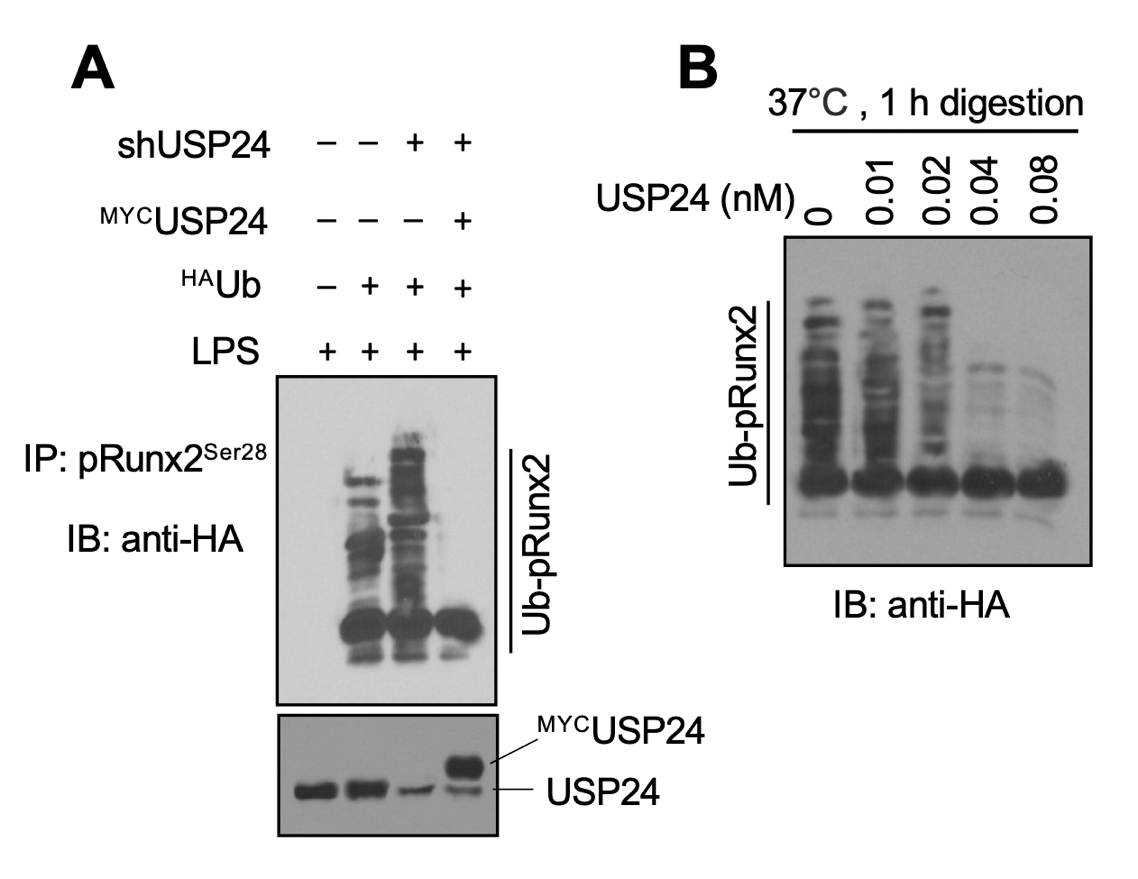


**Figure S9. USP24 deubiquitinated pRunx2 in vivo and in vitro**

**(A)** In vivo ubiquitination assay results. NP-1 cells were infected with the indicated plasmids. After 48 h, cells were treated with 20 ng/mL LPS for 6 h. Cells were collected for IP assay using anti-pRunx2^Ser28^ antibody-coated protein A agarose. Ubiquitination was detected using anti-HA antibody. The levels of USP24 protein in cell extracts before the IP assay were determined. **(B)** USP24-mediated cleavage of ubiquitinated pRunx2 in vitro. Equal amounts of immunoprecipitated Runx2^Ser28^ from LPS-treated NP-1 cells expressing shUSP24 + HA-Ubiquitin were incubated with various concentrations (0, 0.01, 0.02, 0.04, and 0.08 nM) of recombinant USP24 at 37°C for 1 hour. Ubiquitination was detected using anti-HA antibody.

**
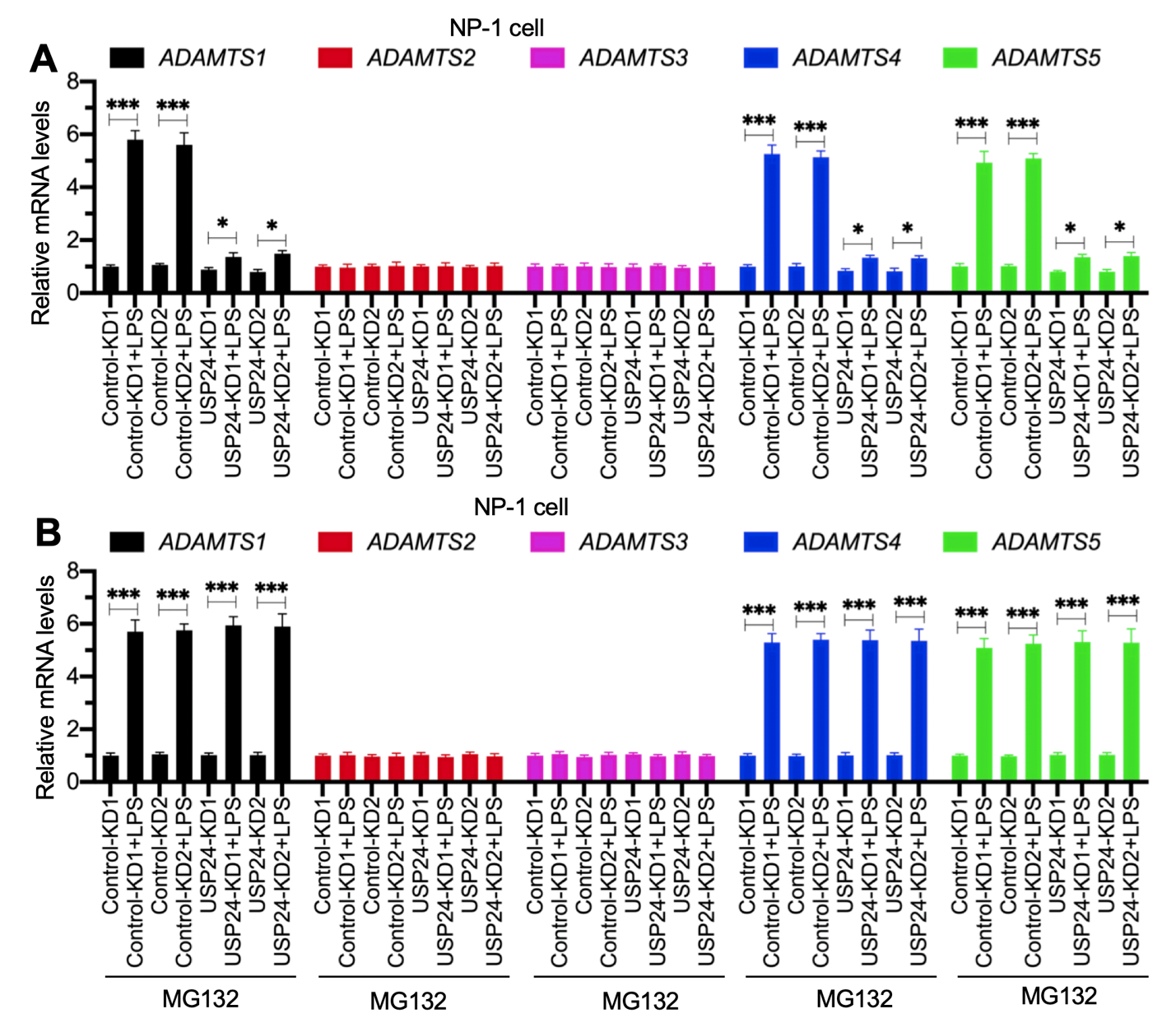
**

**Figure S10. The effects of USP24 depletion and MG132 on the expression levels of *ADAMTS1/2/3/4/5* in NP-1 cells**

**(A)** The mRNA levels of *ADAMTS1/2/3/4/5* in USP24-KD cells treated with LPS. The Control-KD1/2 and USP24-KD1/2 cells in NP-1 background were treated with or without 20 ng/mL LPS for 6 h, followed by RNA isolation and RT-qPCR analyses to examine mRNA levels of *ADAMTS1/2/3/4/5.* **(B)** The mRNA levels of *ADAMTS1/2/3/4/5* in USP24-KD cells co-treated with LPS and MG132. The Control-KD1/2 and USP24-KD1/2 cells in NP-1 background were co-treated with 20 ng/mL LPS and 10 µM MG132 for 6 h, followed by RNA isolation and RT-qPCR analyses to examine mRNA levels of *ADAMTS1/2/3/4/5.* **P* < 0.05; ****P* < 0.001.

**
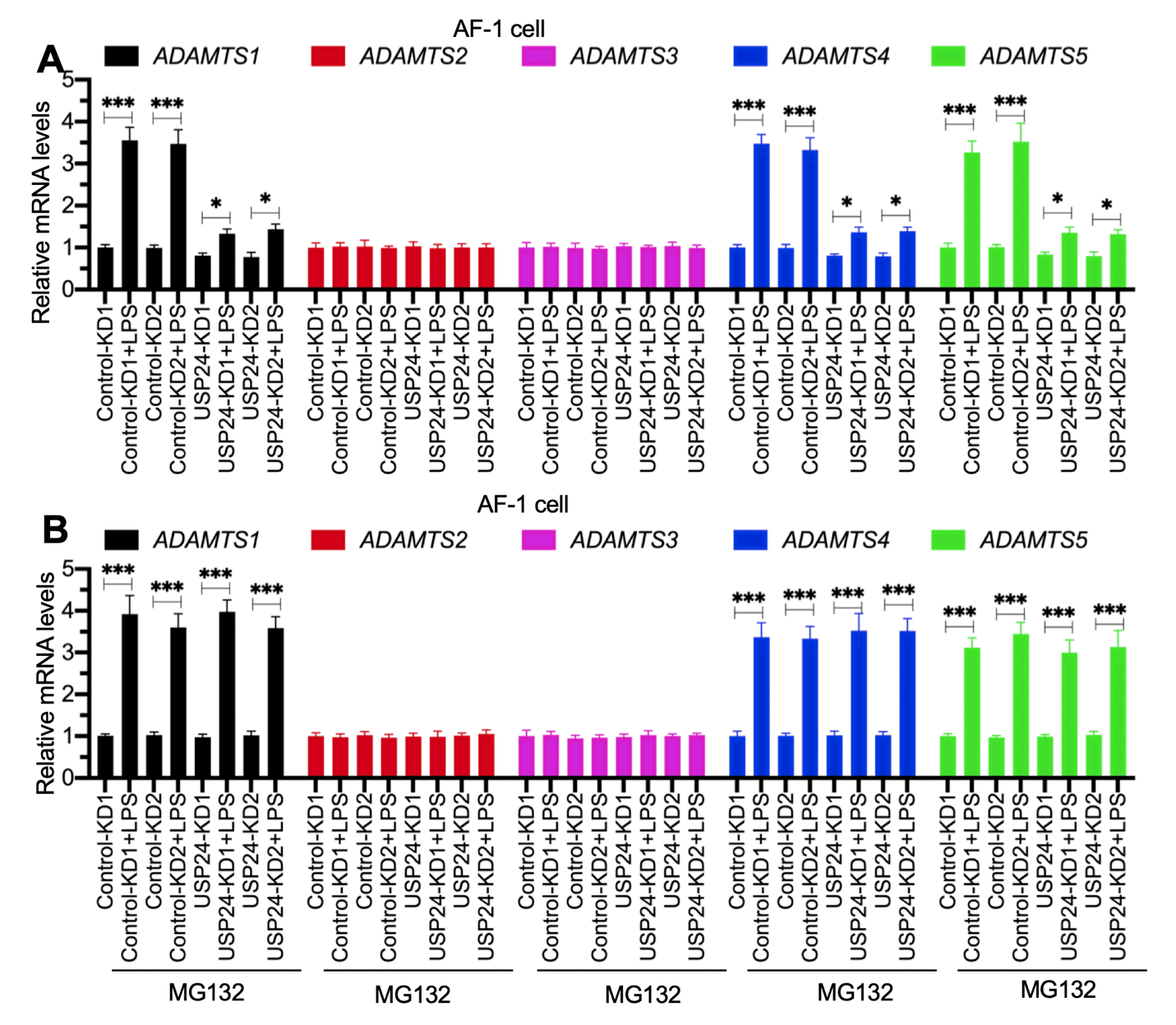
**

**Figure S11. The effects of USP24 depletion and MG132 on the expression levels of *ADAMTS1/2/3/4/5* in AF-1 cells**

**(A)** The mRNA levels of *ADAMTS1/2/3/4/5* in USP24-KD cells treated with LPS. The Control-KD1/2 and USP24-KD1/2 cells in AF-1 background were treated with or without 20 ng/mL LPS for 6 h, followed by RNA isolation and RT-qPCR analyses to examine mRNA levels of *ADAMTS1/2/3/4/5.* **(B)** The mRNA levels of *ADAMTS1/2/3/4/5* in USP24-KD cells co-treated with LPS and MG132. The Control-KD1/2 and USP24-KD1/2 cells in AF-1 background were co-treated with 20 ng/mL LPS and 10 µM MG132 for 6 h, followed by RNA isolation and RT-qPCR analyses to examine mRNA levels of *ADAMTS1/2/3/4/5.* **P* < 0.05; ****P* < 0.001.

**
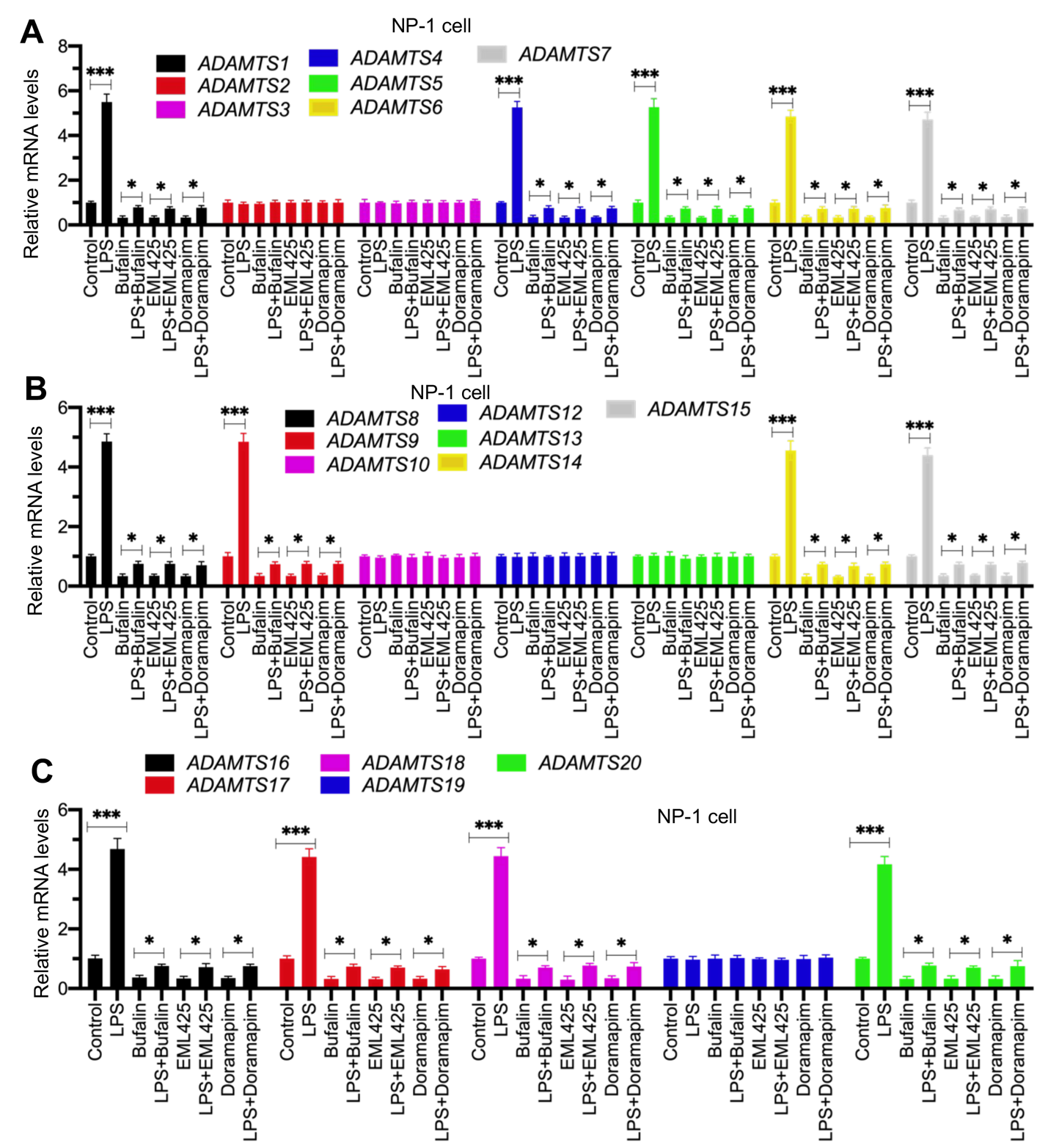
**

**Figure S12. The effects of p38/NCOA3/p300 inhibitors on the expression of *ADAMTSs* in NP-1 cells treated with or without LPS**

The NP-1 cells were treated with an NCOA3 inhibitor Bufalin (10 nM), a p300 inhibitor EML425 (1 μM), or a p38 inhibitor Doramapimod (40 nM) alone or co-treated with 20 ng/mL LPS+10 nM Bufalin, 20 ng/mL LPS+1 μM EML425, 20 ng/mL LPS+40 nM Doramapimod for 6 h, followed by RNA isolation and RT-qPCR analyses to examine mRNA levels of *ADAMTSs*. **(A)** *ADAMTS1*, *-2*, *-3*, *-4*, *-5*, *-6*, and *-7*. **(B)** *ADAMTS8*, *-9, -10, -12,* *-13*, *-14*, and *-15*. **(C)** *ADAMTS16*, *-17*, *-18*, *-19*, and *-20*. **P* < 0.05; ****P* < 0.001.

**
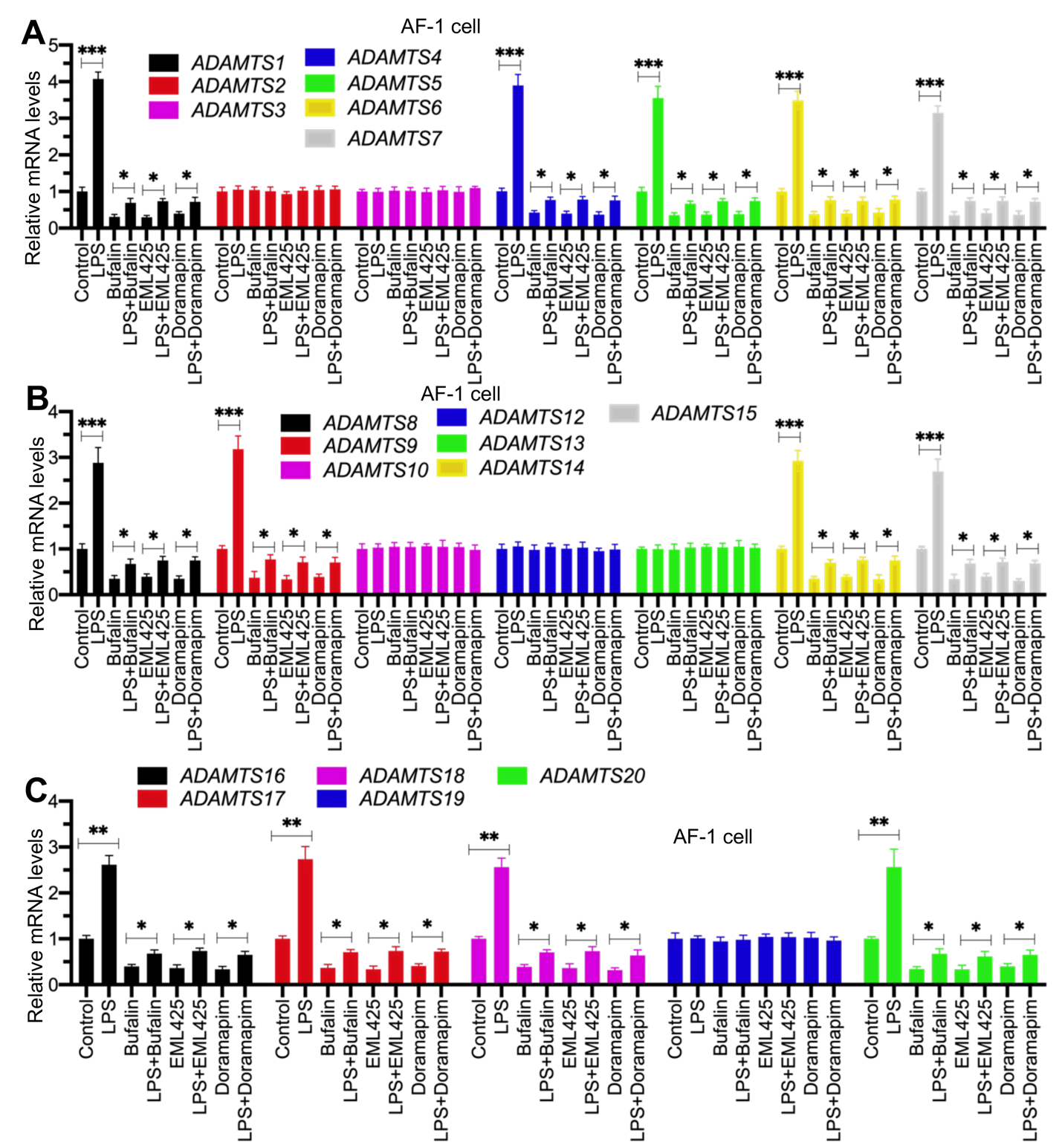
**

**Figure S13. The effects of p38/NCOA3/p300 inhibitors on the expression of *ADAMTSs* in AF-1 cells treated with or without LPS**

The AF-1 cells were treated with an NCOA3 inhibitor Bufalin (10 nM), a p300 inhibitor EML425 (1 μM), or a p38 inhibitor Doramapimod (40 nM) alone or co-treated with 20 ng/mL LPS+10 nM Bufalin, 20 ng/mL LPS+1 μM EML425, 20 ng/mL LPS+40 nM Doramapimod for 6 h, followed by RNA isolation and RT-qPCR analyses to examine mRNA levels of *ADAMTSs*. **(A)** *ADAMTS1*, *-2*, *-3*, *-4*, *-5*, *-6*, and *-7*. **(B)** *ADAMTS8*, *-9, -10, -12,* *-13*, *-14*, and *-15*. **(C)** *ADAMTS16*, *-17*, *-18*, *-19*, and *-20*. **P* < 0.05; ****P* < 0.001.

**
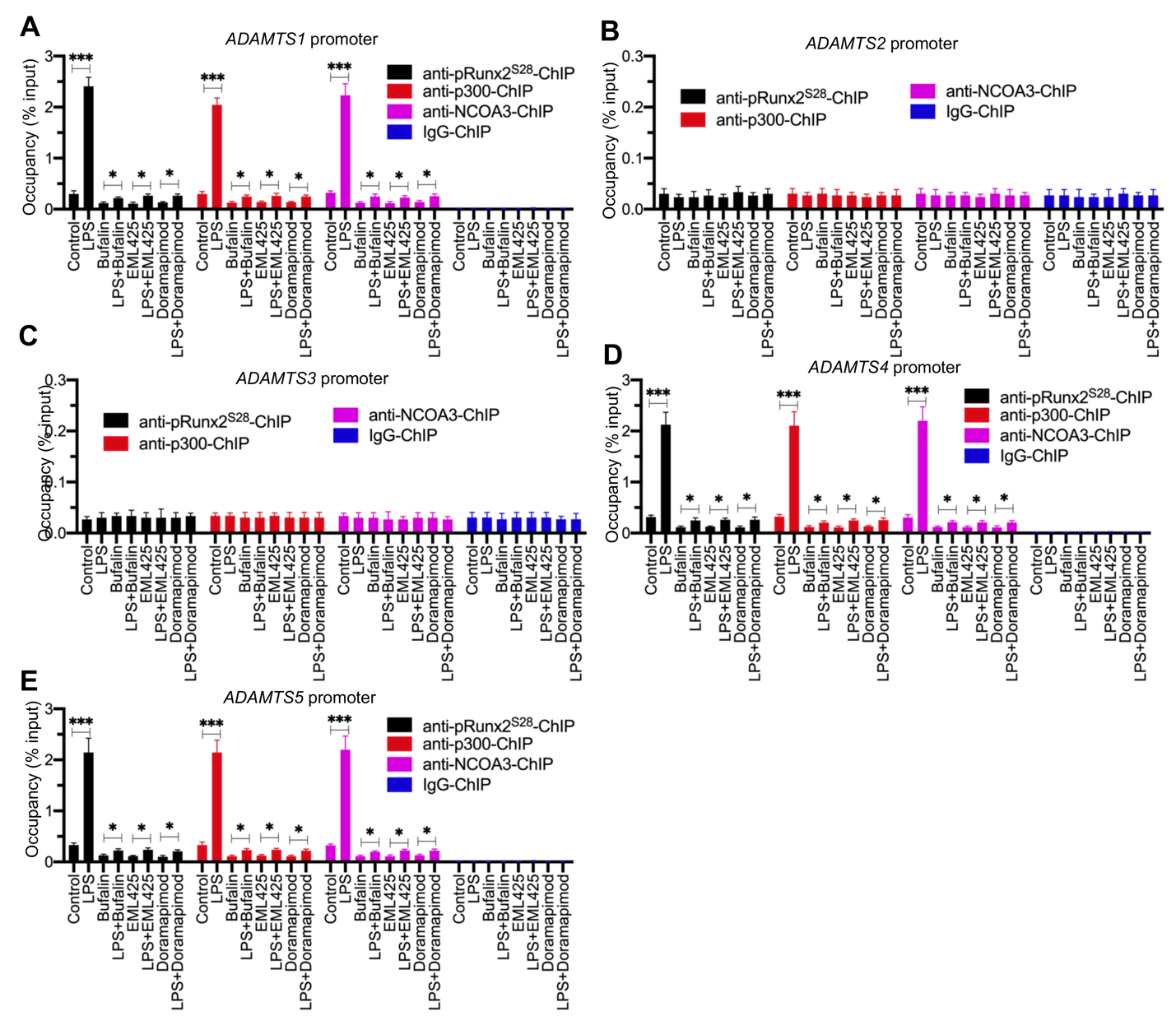
**

**Figure S14. The effects of p38/NCOA3/p300 inhibitors on the occupancies of NCOA3-p300-pRunx2 complex on the promoters of *ADAMTSs* in NP-1 cells**

Cells in Figure S12 were used for ChIP assays with anti-NCOA3-, anti-p300-, anti-pRunx2^S28^-, and IgG-coated protein A agarose. The input and output DNA samples were used for RT-qPCR analyses to examine the occupancies of NCOA3-p300-pRunx2 complex on the promoters of *ADAMTS1* **(A)**, *ADAMTS2* **(B)**, *ADAMTS3* **(C)**, *ADAMTS4* **(D)**, and *ADAMTS5* **(E)**. **P* < 0.05; ****P* < 0.001.

**
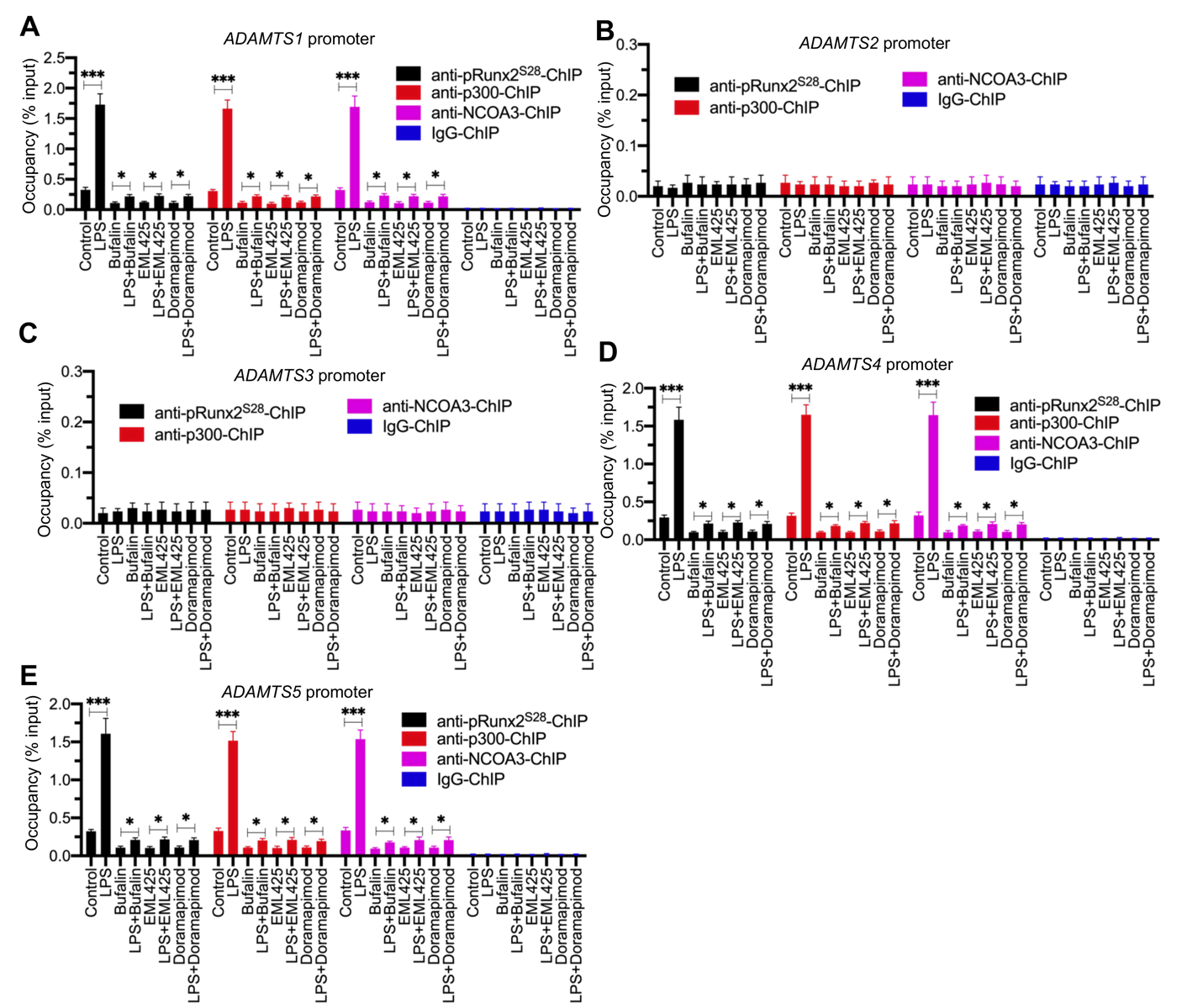
**

**Figure S15. The effects of p38/NCOA3/p300 inhibitors on the occupancies of NCOA3-p300-pRunx2 complex on the promoters of *ADAMTSs* in AF-1 cells**

Cells in Figure S13 were used for ChIP assays with anti-NCOA3-, anti-p300-, anti-pRunx2^S28^-, and IgG-coated protein A agarose. The input and output DNA samples were used for RT-qPCR analyses to examine the occupancies of NCOA3-p300-pRunx2 complex on the promoters of *ADAMTS1* **(A)**, *ADAMTS2* **(B)**, *ADAMTS3* **(C)**, *ADAMTS4* **(D)**, and *ADAMTS5* **(E)**. **P* < 0.05; ****P* < 0.001.


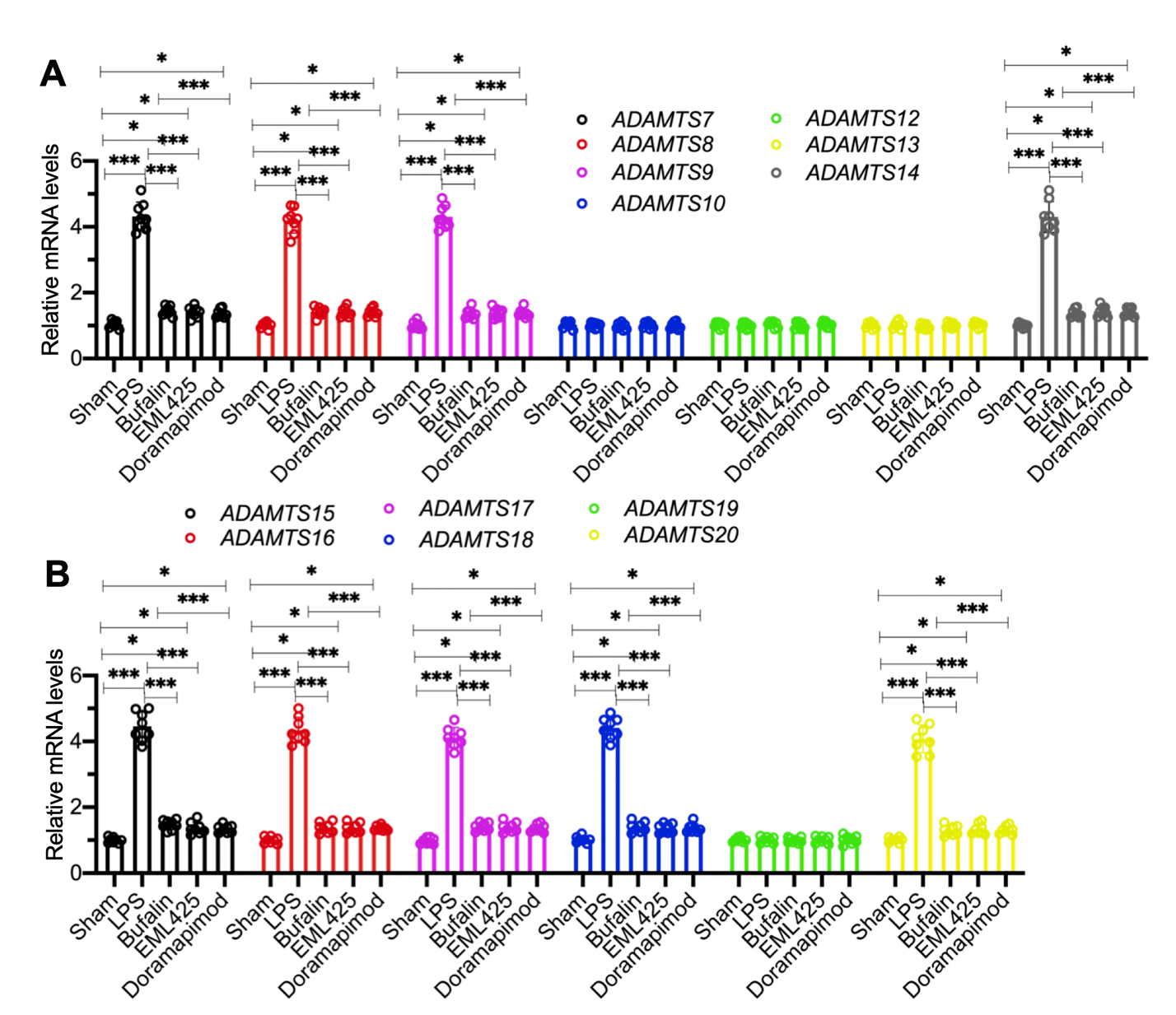


**Figure S16. The mRNA expression levels of *ADAMTSs* in IVDs from mice administrated p38/NCOA3/p300 inhibitors**

The same cDNA samples as shown in Figure 7F were used to detect mRNA levels of *ADAMTSs*. **(A)** *ADAMTS7, -8, -9, 10*, -*12, -13,* and *-14*; **(B)** *ADAMTS15, -16*, -*17, -18, -19,* and -*20*; **P* < 0.05; ****P* < 0.001.

**Table S1. shRNAs and their sources in gene knockdown**

| **Gene** | **TRC Clone ID** | **Sources** |
| --- | --- | --- |
| Runx2 | TRCN0000095590 | Sigma-Aldrich, China |
|  | TRCN0000421148 | Sigma-Aldrich, China |
| p300 | TRCN0000071204 | Sigma-Aldrich, China |
|  | TRCN0000071206 | Sigma-Aldrich, China |
| NCOA3 | TRCN0000095795 | Sigma-Aldrich, China |
|  | TRCN0000095796 | Sigma-Aldrich, China |
| p38 | TRCN0000023119 | Sigma-Aldrich, China |
|  | TRCN0000023120 | Sigma-Aldrich, China |
| USP24 | TRCN0000040628 | Sigma-Aldrich, China |
|  | TRCN0000086744 | Sigma-Aldrich, China |

**Table S2. Plasmids for gene overexpression and co-immunoprecipitation**

| **Vectors** | **Insertion sites** | **Forward primers** | **Reverse primers** |
| --- | --- | --- | --- |
| pCDNA3-Flag-Runx2 | BamHI+XhoI | CGGGATCCATGGCGTCAAACAGCCTCTTC | CCGCTCGAGTCAATATGGCCGCCAAACAG |
| pCDNA3-Flag-Runx2^S28D^ | BamHI+XhoI | CGGGATCCATGGCGTCAAACAGCCTCTTC | CCGCTCGAGTCAATATGGCCGCCAAACAG |
| pCDNA3-Flag-p300 | BamHI+XhoI | CGGGATCCATGGCCGAGAATGTGGTGGAA | CCGCTCGAGCAGCTTGTTGAGGCAGAGTAG |
| pCDNA3-Myc-p300 | BamHI+XhoI | CGGGATCCATGGCCGAGAATGTGGTGGAA | CCGCTCGAGCAGCTTGTTGAGGCAGAGTAG |
| pCDNA3-Myc-NCOA3 | BamHI+XhoI | CGGGATCCATGAGTGGACTAGGCGAAAG | CCGCTCGAGTCAGCAGTATTTCTGATCGGG |
| pCDNA3-Myc-Runx2 | BamHI+XhoI | CGGGATCCATGGCGTCAAACAGCCTCTTC | CCGCTCGAGTCAATATGGCCGCCAAACAG |
| pCDNA3-Myc-USP24 | BamHI+XhoI | CGGGATCCATGGAATCGGAGGAGGAGCA | CCGCTCGAGCTAAGGATCAACATCAT |

**Table S3. Primers for RT-qPCR to detect gene expression levels**

| **Genes** | **Forward primers (5’-3’)** | **Reverse primers (5’-3’)** |
| --- | --- | --- |
| ADAMTS1 | GACACCCTGCTTCCGAATGTG | AGGTTCTCTTCTTGTAACCCTTC |
| ADAMTS2 | CCTCATCAACTGTAGATCTA | CACACACACAAGTTCACACAAG |
| ADAMTS3 | CCATCAGACGCATGTGCAACA | GGTTGGTGCCATCCTGCAGTG |
| ADAMTS4 | GAGCAGTGTGCTGCCTACAAC | GCTCCAATACGTAGTAGTAG |
| ADAMTS5 | GATCCTAGCTTACATGTGATC | TTCAGGTGCTGCTCCCAGGCAG |
| ADAMTS6 | TGTCGTTTCCAGTATGGAG | ATATTCCCAGTCTGACACAGTG |
| ADAMTS7 | TCTTACCTGGTCCCGATGCAG | CACTGGTGGTTCACATCATAC |
| ADAMTS8 | ACAGCACCCTCTACGAGCTG | TGGCAAATGGGCTCATCACTAT |
| ADAMTS9 | TATATGATGCAGTGCAGACGG | CACAAAATCCAAACTTGCAGT |
| ADAMTS10 | TCTGCAGTGAACTGTGGTGTC | ACACCCTCTGGCCGAGAGCCA |
| ADAMTS12 | CACCAACGAGTCAGTGTGGCT | TGCATTCTGTCCAGCGGCCAA |
| ADAMTS13 | TGTGATGGCAGGATGGACTC | GGAGTAACAATCAGGAACGT |
| ADAMTS14 | ATCACGACCACGTCATCTTCC | TGTCTCATGAGCCACGACAA |
| ADAMTS15 | TCACTTTAGCTGTGGCATGGG | TCAGGAGTACACAGCGTACCAT |
| ADAMTS16 | GATCAGATGCAACAGAGGAC | TGAATGCTCCGAGCTCCAGAA |
| ADAMTS17 | AGCTGCCAGGCATGCACTAC | CTTGGTCTTGCAGGATGTGTC |
| ADAMTS18 | GAACCATGTGACACACTAGGA | ACCATCATGAACCATGCCAA |
| ADAMTS19 | GATGATGACAGAATGGACCA | CACAGTTGCGGGCTTTGGCC |
| ADAMTS20 | ATCGCTGTAAACTCTATTGCC | ACACGTGATCACAGCCAGCT |
| Runx2 | CTATCTGAGCCAGATGACATC | AGTCAGAGGTGGCAGTGTC |
| P300 | CACATCCCAAGGAATGAATG | AGCTTGTTGAGGCAGAGTAG |
| NCOA3 | CGAATGATCGTCACGGCTTC | ATTTGGAGACATGCTCACG |
| P38 | ATCACAGCAGCCCAAGCT | CTTCATCATAGGTCAGGCT |
| USP24 | TGCTTTGCATGCGCTTCCAAC | AGACTTGGATAGAGTACTATC |
| Beta-Actin | ATTGGCAACGAGCGGTTCCG | AGCACTGTGTTGGCATAGAGG |

**Table S4. Primary and secondary antibodies for western blotting assays**

| **Antibodies** | **Catalog numbers** | **Dilution folds** | **Sources** |
| --- | --- | --- | --- |
| Anti-Runx2 | #PA5-86506 | 1:3000 | Thermo Fisher |
| Anti-NCOA3 | #2126 | 1:2500 | Cell signaling |
| Anti-p300 | #54062 | 1:2000 | Cell signaling |
| Anti-p38 | #ab170099 | 1:4000 | Abcam |
| Anti-pRunx2^S28^ | #PA5-105643 | 1:3500 | Thermo Fisher |
| Anti-pRunx2^S275^ | #PA5-105642 | 1:3000 | Thermo Fisher |
| Anti-pRunx2^S340^ | #PA5-105209 | 1:3000 | Thermo Fisher |
| Anti-USP24 | #SAB1300264 | 1:3500 | Sigma-Aldrich |
| Anti-MYC | #ab32 | 1:5000 | Abcam |
| Anti-Flag | #ab236777 | 1:5000 | Abcam |
| Anti-HA | #11583816001 | 1:6000 | Sigma-Aldrich |
| Goat Anti-Rabbit IgG | #ab205718 | 1:10000 | Abcam |
| Goat Anti-Mouse IgG | #ab205719 | 1:10000 | Abcam |
| Anti-GAPDH | #ab8245 | 1:5000 | Abcam |

**Table S5. Primers for ChIP RT-qPCR**

| **Gene promoters** | **Forward primers (5’-3’)** | **Reverse primers (5’-3’)** |
| --- | --- | --- |
| ADAMTS1 | AGCCATGGTGCCCATGGA | AAGAGGGTCGAATTGAA |
| ADAMTS2 | CAGAATGATCGTGTCTTCA | GCCAAAATGAGCCCACAT |
| ADAMTS3 | CAAGACCTGACTCCACT | AGTTCTCCTATCCTGGA |
| ADAMTS4 | CAGCTTGGCTCTAACAGA | CACAGATGAGACTCTGTCCT |
| ADAMTS5 | TTTCAGGATCCATTCAG | GGCCAAGACTGGAGAA |

**Table S6. The potential Runx2-interacting proteins identified by mass spectrometry**

| **Proteins** | **Protein description** | **Molecular weight (kDa)** | **MASCOT scores** |
| --- | --- | --- | --- |
| Runx2 | Runt-Related Transcription Factor 2 | 57 | 1816 |
| P300 | Histone Acetyltransferase P300 | 265 | 1805 |
| NCOA3 | Nuclear Receptor Coactivator 3 | 155 | 1774 |
| BGLAP | Bone Gamma-Carboxyglutamate Protein | 11 | 1713 |
| DDX5 | DEAD-Box Helicase 5 | 69 | 1662 |
| BMP4 | Bone Morphogenetic Protein 4 | 47 | 1631 |
| VDR | Vitamin D Receptor | 49 | 1558 |
| CCND1 | Cyclin D1 | 34 | 1522 |
| XPO1 | Exportin 1 | 123 | 1448 |
| NDST1 | N-Deacetylase And N-Sulfotransferase 1 | 101 | 1421 |
| PBDC1 | Polysaccharide Biosynthesis Domain Containing 1 | 26 | 1403 |
| INTS9 | Integrator Complex Subunit 9 | 74 | 1384 |
| GPC6 | Glypican 6 | 63 | 1377 |
| GAD1 | Glutamate Decarboxylase 1 | 67 | 1325 |
| EDF1 | Endothelial Differentiation Related Factor 1 | 16 | 1311 |
| PRDX6 | Peroxiredoxin 6 | 25 | 1227 |
| ACAT2 | Acetyl-CoA Acetyltransferase 2 | 41 | 1205 |
| NUDT2 | Nudix Hydrolase 2 | 17 | 1187 |
| TMC1 | Transmembrane Channel Like 1 | 88 | 1165 |
| PLXDC2 | Plexin Domain Containing 2 | 60 | 1142 |
| VPS25 | Vacuolar Protein Sorting 25 Homolog | 21 | 1101 |
| LCMT2 | Leucine Carboxyl Methyltransferase 2 | 76 | 987 |
| STIM1 | Stromal Interaction Molecule 1 | 77 | 963 |
| RING1 | Ring Finger Protein 1 | 42 | 922 |
| POLD3 | DNA Polymerase Delta 3, Accessory Subunit | 51 | 847 |
| SOCS4 | Suppressor Of Cytokine Signaling 4 | 51 | 823 |
| MED1 | Mediator Complex Subunit 1 | 168 | 802 |
| MBD2 | Methyl-CpG Binding Domain Protein 2 | 43 | 789 |
| NR5A2 | Nuclear Receptor Subfamily 5 Group A Member 2 | 61 | 771 |
| DNM3 | Dynamin 3 | 98 | 723 |
| ABCF2 | ATP Binding Cassette Subfamily F Member 2 | 71 | 669 |
| XBP1 | X-Box Binding Protein 1 | 29 | 654 |
| MYOD1 | Myogenic Differentiation 1 | 35 | 634 |
| PRMT1 | Protein Arginine Methyltransferase 1 | 42 | 611 |
| PRS16 | Ribosomal Protein S16 | 16 | 603 |
| CHD9 | Chromodomain Helicase DNA Binding Protein 9 | 326 | 587 |
| GMEB2 | Glucocorticoid Modulatory Element Binding Protein 2 | 56 | 554 |
| NOL11 | Nucleolar Protein 11 | 81 | 521 |
| PLCG1 | Phospholipase C Gamma 1 | 149 | 505 |
| GNB5 | G Protein Subunit Beta 5 | 44 | 485 |
| HMGB2 | High Mobility Group Box 2 | 24 | 442 |
| NOS3 | Nitric Oxide Synthase 3 | 133 | 407 |

**Table S7. The potential pRunx2S^28^-interacting proteins identified by mass spectrometry**

| **Proteins** | **Protein description** | **Molecular weight (kDa)** | **MASCOT scores** |
| --- | --- | --- | --- |
| Runx2 | Runt-Related Transcription Factor 2 | 57 | 2077 |
| USP24 | Ubiquitin Specific Peptidase 24 | 294 | 2025 |
| BRD2 | Bromodomain Containing 2 | 88 | 1884 |
| BRD7 | Bromodomain Containing 7 | 74 | 1763 |
| GMEB2 | Glucocorticoid Modulatory Element Binding Protein 2 | 56 | 1543 |
| MED1 | Mediator Complex Subunit 1 | 168 | 1522 |
| PLCG1 | Phospholipase C Gamma 1 | 149 | 1428 |
| MBD2 | Methyl-CpG Binding Domain Protein 2 | 43 | 1277 |
| XPO1 | Exportin 1 | 123 | 1205 |
| LCMT2 | Leucine Carboxyl Methyltransferase 2 | 76 | 1146 |
| VPS25 | Vacuolar Protein Sorting 25 Homolog | 21 | 1108 |
| CCDC15 | Coiled-Coil Domain Containing 15 | 110 | 1028 |
| SIAE | Sialic Acid Acetylesterase | 58 | 954 |
| WDR20 | WD Repeat Domain 20 | 63 | 948 |
| GGT1 | Gamma-Glutamyltransferase 1 | 61 | 911 |
| SPP1 | Secreted Phosphoprotein 1 | 35 | 902 |
| SOCS4 | Suppressor Of Cytokine Signaling 4 | 51 | 854 |
| NUDT2 | Nudix Hydrolase 2 | 17 | 826 |
| RAG1 | Recombination Activating 1 | 119 | 801 |
| RAG2 | Recombination Activating 2 | 59 | 779 |
| DCAF1 | DDB1 And CUL4 Associated Factor 1 | 169 | 753 |
| PCGF3 | Polycomb Group Ring Finger 3 | 28 | 639 |
| MAGT1 | Magnesium Transporter 1 | 38 | 622 |
| NOL11 | Nucleolar Protein 11 | 81 | 601 |
| NOS3 | Nitric Oxide Synthase 3 | 133 | 549 |
| PRDX6 | Peroxiredoxin 6 | 25 | 522 |
| DNM3 | Dynamin 3 | 98 | 487 |
| ABCF2 | ATP Binding Cassette Subfamily F Member 2 | 71 | 449 |
| PGM3 | Phosphoglucomutase 3 | 60 | 435 |
| NRG4 | Neuregulin 4 | 13 | 422 |
| NR5A2 | Nuclear Receptor Subfamily 5 Group A Member 2 | 61 | 408 |
